# Epidemiological analysis of maternal hypertensive disorders of pregnancy

**DOI:** 10.3389/fmed.2025.1498694

**Published:** 2025-08-01

**Authors:** Aijuan Yuan, Hui Huang

**Affiliations:** Department of Obstetrics and Gynecology, Ningbo University Affiliated People’s Hospital, Zhejiang, China

**Keywords:** maternal hypertensive disorders, incidence, estimated annual percentage change, disability-adjusted life years, global burden of disease

## Abstract

**Background:**

We aimed to provide a comprehensive overview of the epidemiology of hypertensive disorders of pregnancy (HDP) from 1990 to 2021, examining incidence and trends across national, continental, and global levels.

**Methods:**

HDP were assessed by calculating the age-standardized incidence rate (ASIR), age-standardized disability-adjusted life years (DALYs) rate, and the estimated annual percentage change (EAPC) for each region and country. The Global Burden of Disease (GBD) 2021 Study age-standardized rates, which were obtained from 204 countries and territories, were used to analyze the burden based on age and sociodemographic index (SDI).

**Results:**

The global burden of HDP has changed substantially between 1990 and 2021. The total number of DALYs was 2.47 million (95% UI: 2.08 to 2.96), and the ASIR was 461.94 per 100,000 in 2021. The estimated annual percentage change (EAPC) for the DALY rate was −2.10 (95% CI: −2.15 to −2.04), and the EAPC for ASIR was −0.51 (95% CI: −0.56 to −0.45). The Russian Federation, Romania, and Georgia had the greatest increases in ASIR over time. Guam, American Samoa, and the United Republic of Tanzania had the highest EAPC for the DALY rate. Analysis by age showed that maternal hypertensive disorders caused the highest DALYs among women aged 25–39 years and the lowest DALYs among those aged 50–54 years. There was a positive relationship between ASIR and SDI. The same trend was observed for the DALY rate.

**Conclusion:**

Our findings revealed that DALY rates and incidence rates of HDP decreased in most regions, except in areas with lower sociodemographic indices, such as the Caribbean. The greatest burden of HDP was observed among women aged 25–39 years. These results underscore the need for targeted interventions in low-SDI regions and highlight the importance of addressing HDP as a key component of maternal health initiatives globally.

## 1 Introduction

Hypertensive disorders of pregnancy (HDP) are a primary contributor to global maternal and perinatal morbidity and mortality (1). The 2013 guidelines from the American Congress of Obstetricians and Gynecologists categorize HDP into four distinct types: gestational hypertension, preeclampsia/eclampsia, chronic hypertension, and chronic hypertension with superimposed preeclampsia/eclampsia (2). The 2020 guidelines from the International Society of Hypertension retain this classification system while additionally identifying hemolysis, elevated liver enzymes, and low platelets (HELLP) syndrome, characterized by hemolytic anemia, elevated liver enzymes, and low platelet count as a unique entity (3). Regardless of the classification, monitoring blood pressure during pregnancy remains essential.

Evidence indicates that HDP are correlated with significant long-term health hazards for both women and their offspring (4). Effective management of HDP can reduce mortality and mitigate the adverse effects of preeclampsia, which is associated with severe maternal complications such as premature labor, prolonged hospitalization, and psychological distress, often leading to disrupted maternal–infant bonding and family separation (5). Furthermore, preeclampsia and gestational hypertension increase the risk of recurrence in subsequent pregnancies (6), future cardiovascular diseases (7), and type 2 diabetes (8–12). Beyond maternal health, HDP also poses risks to fetal and child health. Studies have shown that children born to mothers with HDP are at a higher risk of preterm birth, low birth weight, and long-term developmental issues, including neurodevelopmental delays and an increased susceptibility to chronic conditions such as hypertension and metabolic disorders later in life (13, 14). The impact of HDP extends to the timing and method of delivery, duration of hospital stay, and the mother’s connection with the newborn and family (15), with ramifications that can persist into adulthood for both the mother and the child. These findings underscore the critical importance of addressing HDP not only as a maternal health issue but also as a determinant of fetal and child health outcomes.

Recent studies have reported an increase in the incidence of HDP in recent decades (13, 15), highlighting its emerging importance as a cardiovascular disease affecting women (14). Even though HDP are important for risk assessment and prevention, their epidemiology is largely unknown, especially in developing countries, thereby affecting prevention and healthcare practices. The prevalence of HDP is between 4 and 25%, and it is a major contributor to maternal morbidity and mortality worldwide (14). The rates of preeclampsia and maternal mortality have markedly decreased over the past half-century in developed regions but remain high in developing regions (16). A meta-analysis showed that the global incidence of preeclampsia was 4.6% (95% CI, 2.7–8.2), and the incidence was 1.0 and 5.6% in the Eastern Mediterranean and Africa, respectively (16). This discrepancy makes it difficult to assess the global burden of HDP and hinders efforts to prevent HDP among pregnant women in different parts of the world (17, 18).

This research aims to provide a comprehensive and updated analysis of the epidemiology of HDP by utilizing the latest Global Burden of Diseases (GBD) 2021 data, spanning from 1990 to 2021. Unlike previous studies that were limited to data up to 2019 and focused on specific countries or regions, this study offers a broader global perspective, assessing HDP incidence, disability-adjusted life years (DALYs), and estimated annual percentage change (EAPC) across different age groups and sociodemographic indices (SDI). The purpose of this analysis is to identify temporal trends, regional disparities, and high-risk populations, thereby addressing critical gaps in understanding the global burden of HDP. Such insights are essential for informing public health policies, guiding resource allocation, and designing targeted interventions to reduce maternal morbidity and mortality associated with HDP.

## 2 Method

### 2.1 Data collection

The GBD study offers an exhaustive aggregation of epidemiological data, encapsulating the prevalence, incidence, mortality, years lived with disability (YLDs), DALYs, and years of life lost (YLLs) for 286 causes of death, 369 diseases and injuries, and 87 risk factors across 204 countries and territories (19–21). The data are stratified by sex, age, and year. This study extracted and analyzed annual data concerning the incidence, DALYs, and EAPC of lower respiratory infections from 1990 to 2021. Data were clustered by country/region and SDI quintiles, a measure that represents levels of social development and is associated with total fertility rate, per capita income, and average years of education (22). The data specific to lower respiratory infections were further segmented to elucidate disparities in social development among various countries, regions, and SDI quintiles. As this study employs a publicly accessible database, ethical approval is not required.

### 2.2 Definition

HDP are a heterogeneous group of conditions that include gestational hypertension and preeclampsia. Preeclampsia is characterized by the new onset of hypertension after 20 weeks of pregnancy in women with previously normal blood pressure, in conjunction with proteinuria. It may be complicated by seizures (eclampsia), severe liver disease, low platelet count, and organ damage. Chronic hypertension is defined by the presence of hypertension before pregnancy or of an elevated blood pressure before 20 weeks of gestation that persists for more than 12 weeks postpartum. Chronic hypertension may be complicated by superimposed preeclampsia, which is characterized by the new onset of proteinuria or a rapid increase in blood pressure and symptoms. HELLP syndrome is a variant of severe preeclampsia characterized by hemolysis, elevated liver enzyme levels, and low platelet count.

### 2.3 Statistical analysis

Age-standardized rates (ASRs) and 95% confidence intervals (CIs) were computed based on the world standard population from the 2021 GBD Study. The results are expressed per 100,000 population, and age-standardization was conducted using the method recommended by the World Health Organization (WHO).


$$\mathrm{Age}-\text{standardized rate}=\frac{\sum_{i}^{A}a_{i}w_{i}}{\sum_{i}^{A}w_{i}}\times 100,000$$

where *a_i_* is the age-specific rate, and *w_i_* is the weight of the corresponding age group in the chosen standard population (where *i* denotes the *i*-th age group).

EAPC, a standard indicator of trends in ASRs by regression analysis (23), was defined as the average percentage change in the ASR per year over time intervals. To calculate the EAPC, we used linear regression (*y* = *α* + *βx* + *ε*), where *y* = ln (ASR) and *x* = calendar year. The EAPC was estimated as 100 × [exp(*β*) – 1], and the 95% CI was calculated based on the regression model. Uncertainty intervals (UIs) for each incidence and DALY number were calculated from 1,000 draws as the 2.5th and 97.5th percentiles.

## 3 Results

### 3.1 Global region distribution in the burden of HDP (1990–2021)

**Figure 1:**
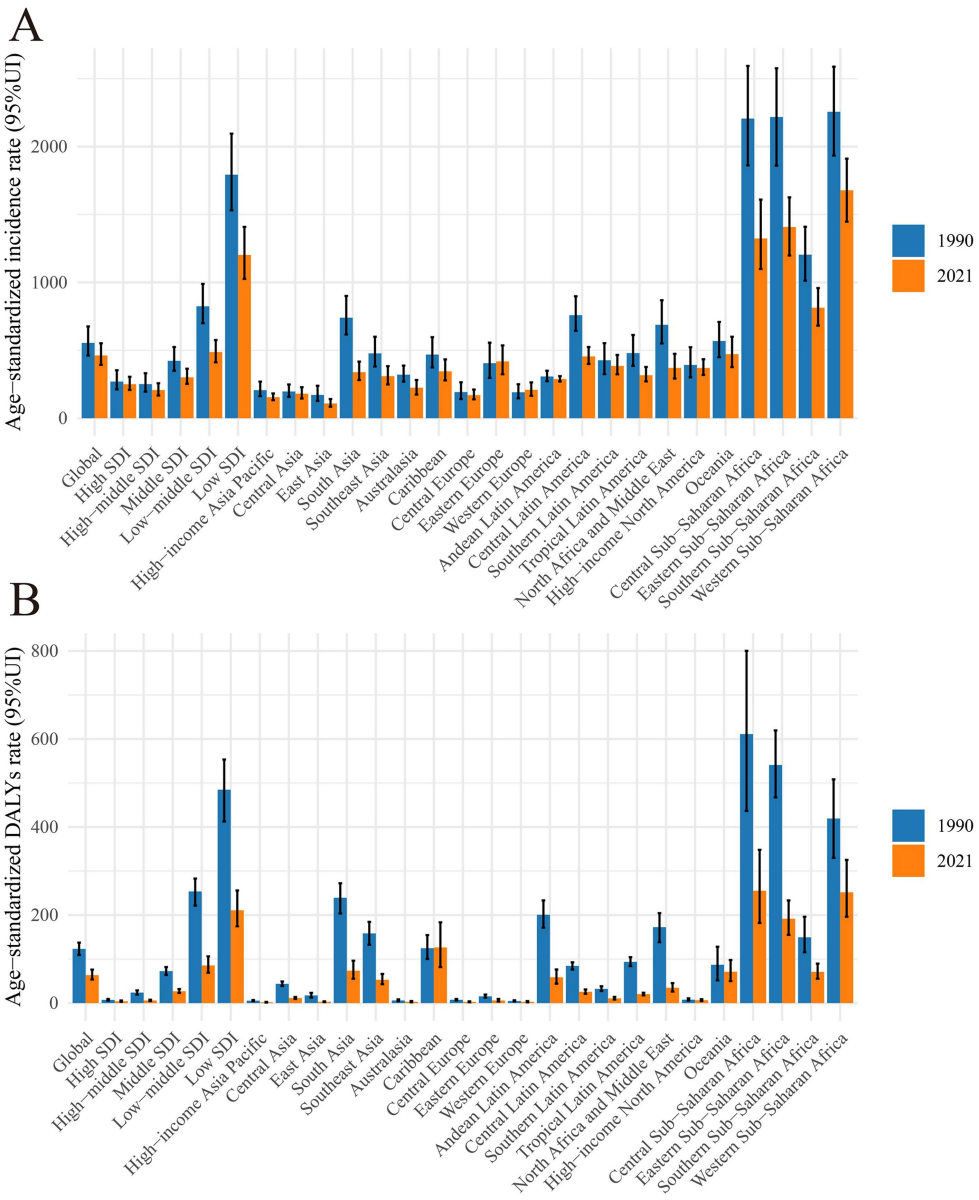
Global burden of maternal hypertensive disorders in 1990 and 2021 by regions. **(A)** Age-standardized incidence rate in 1990 and 2021. **(B)** Age-standardized DALY rate in 1990 and 2021. DALY, disability adjusted life-year.

**Figure 2:**
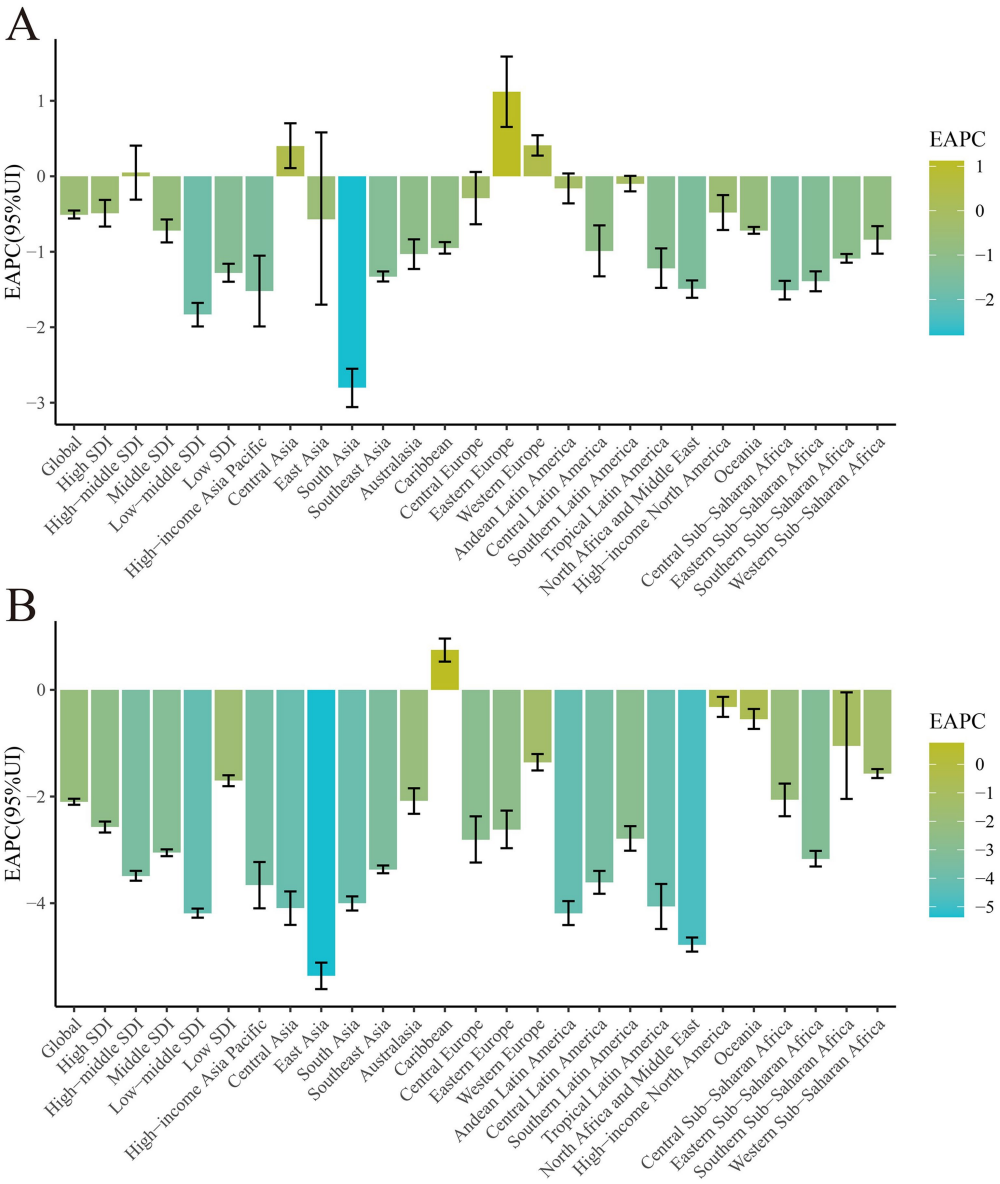
Global burden trend of ASIR and age-standardized DALY rates of maternal hypertensive disorders from 1990 to 2021 by SDI regions. DALY, disability adjusted life-year; ASIR, age-standardized incidence rate.

The global burden of HDP has significantly decreased from 1990 to 2021 (Supplementary Figures 1, 2). Specifically, the global DALYs attributed to maternal hypertensive disorders dropped from 3.48 million (95% UI: 3.09 to 3.87) in 1990 to 2.47 million (95% UI: 2.08 to 2.96) in 2021. Concurrently, the global incidence rate witnessed a reduction, with the ASIR decreasing from 554.35 per 100,000 persons (95% UI: 461.38 to 675.43) in 1990 to 461.94 per 100,000 persons (95% UI: 392.73 to 551.65) in 2021 (Table 1 and Figure 1). The EAPC for both age-standardized DALYs rate and ASIR were −2.10 (95% CI: −2.15 to−2.04) and −0.51 (−0.56, −0.45), respectively, indicating a consistent decline over the three decades (Figure 2).

**Table 1:** Global burden of age-standardized DALY rates and ASIR of maternal hypertensive disorders in 1990, 2021, and EAPC in different regions of the world.

Location	DALYs (disability-adjusted life years)		EAPC	Incidence		EAPC
	1990	2021	1990–2021	1990	2021	1990–2021
	ASR	ASR	EAPC_1990–2021	ASR_1990	ASR_2021	EAPC_1990–2021
Global	123.15 (109.20, 137.37)	63.47 (53.55, 76.02)	−2.10 (−2.1547, −2.0413)	554.35 (461.38, 675.43)	461.94 (392.73, 551.65)	−0.51 (−0.5590, −0.4538)
High SDI	7.25 (5.69, 9.32)	4.18 (2.91, 5.92)	−1.70 (−1.8060, −1.6011)	269.60 (211.65, 353.21)	250.31 (208.38, 304.89)	−0.49 (−0.6662, −0.3134)
High-middle SDI	23.84 (19.22, 28.91)	5.97 (4.66, 7.62)	−4.19 (−4.2716, −4.1013)	251.01 (195.55, 331.19)	207.21 (166.86, 257.15)	0.05 (−0.3098, 0.4060)
Middle SDI	72.63 (63.89, 81.83)	26.84 (23.07, 31.91)	−3.05 (−3.1182, −2.9904)	422.36 (348.93, 524.25)	300.80 (252.74, 364.43)	−0.72 (−0.8761, −0.5722)
Low-middle SDI	253.59 (221.40, 282.92)	85.54 (69.11, 106.12)	−3.49 (−3.5784, −3.3935)	823.86 (699.94, 989.06)	487.24 (411.49, 575.58)	−1.83 (−1.9897, −1.6773)
Low SDI	484.86 (412.55, 553.38)	210.89 (173.87, 255.87)	−2.57 (−2.6747, −2.4691)	1792.92 (1531.06, 2095.17)	1202.32 (1025.84, 1408.89)	−1.28 (−1.3970, −1.1591)
Andean Latin America	200.70 (171.36, 233.29)	58.68 (44.00, 76.40)	−4.19 (−4.4097, −3.9609)	307.22 (271.68, 349.51)	288.22 (270.01, 309.68)	−0.16 (−0.3590, 0.0375)
Australasia	6.01 (4.32, 8.44)	2.97 (1.82, 4.70)	−2.08 (−2.3239, −1.8450)	320.59 (269.02, 387.38)	224.57 (173.84, 281.37)	−1.03 (−1.2283, −0.8355)
Caribbean	124.67 (100.30, 154.48)	126.37 (81.88, 183.60)	0.75 (0.5296, 0.9614)	468.30 (373.93, 596.89)	345.10 (278.18, 433.02)	−0.95 (−1.0247, −0.8722)
Central Asia	43.80 (38.88, 49.13)	11.46 (9.48, 13.84)	−4.09 (−4.4073, −3.7793)	196.58 (156.98, 248.24)	180.35 (144.84, 228.23)	0.40 (0.1082, 0.7022)
Central Europe	7.46 (6.14, 9.27)	2.72 (1.87, 3.95)	−2.81 (−3.2374, −2.3715)	192.52 (140.98, 264.48)	169.62 (138.88, 210.94)	−0.29 (−0.6356, 0.0570)
Central Latin America	84.50 (76.19, 92.66)	25.67 (21.19, 30.95)	−3.61 (−3.8228, −3.3945)	758.62 (643.30, 897.84)	455.00 (399.42, 524.36)	−0.99 (−1.3256, −0.6504)
Central Sub-Saharan Africa	611.23 (436.47, 800.29)	255.22 (182.06, 348.26)	−2.06 (−2.3695, −1.7577)	2206.02 (1861.35, 2593.78)	1323.57 (1098.63, 1609.63)	−1.51 (−1.6319, −1.3856)
East Asia	17.50 (12.54, 23.22)	2.97 (2.18, 4.01)	−5.36 (−5.6106, −5.1146)	171.41 (127.45, 239.11)	108.74 (84.21, 140.96)	−0.57 (−1.7006, 0.5812)
Eastern Europe	15.34 (12.57, 19.32)	5.93 (3.63, 9.30)	−2.62 (−2.9689, −2.2637)	405.04 (296.85, 555.96)	417.91 (323.56, 535.96)	1.12 (0.6532, 1.5859)
Eastern Sub-Saharan Africa	540.94 (467.39, 619.53)	191.51 (155.03, 233.27)	−3.17 (−3.3118, −3.0190)	2217.74 (1859.57, 2576.39)	1408.09 (1198.03, 1626.08)	−1.39 (−1.5234, −1.2594)
High-income Asia Pacific	5.47 (4.33, 6.91)	1.90 (1.30, 2.74)	−3.66 (−4.0963, −3.2282)	206.53 (162.41, 269.35)	154.28 (132.33, 182.35)	−1.52 (−1.9896, −1.0518)
High-income North America	7.71 (5.68, 10.78)	6.57 (4.70, 8.81)	−0.32 (−0.5066, −0.1313)	392.14 (300.76, 522.67)	369.83 (318.70, 434.19)	−0.48 (−0.7121, −0.2493)
North Africa and the Middle East	172.25 (138.43, 204.40)	34.77 (26.20, 45.35)	−4.78 (−4.9074, −4.6430)	687.79 (550.29, 868.70)	370.18 (291.85, 474.07)	−1.49 (−1.6100, −1.3796)
Oceania	87.07 (51.57, 128.05)	71.19 (49.91, 97.87)	−0.55 (−0.7322, −0.3587)	568.38 (449.38, 708.83)	471.69 (376.23, 599.70)	−0.72 (−0.7615, −0.6710)
South Asia	239.29 (203.52, 272.23)	73.56 (55.16, 96.28)	−4.00 (−4.1369, −3.8722)	740.13 (616.22, 900.44)	338.92 (280.67, 416.78)	−2.80 (−3.0578, −2.5501)
Southeast Asia	158.33 (132.51, 184.49)	52.81 (42.93, 66.17)	−3.37 (−3.4385, −3.2927)	477.34 (380.83, 599.77)	309.44 (248.43, 383.27)	−1.33 (−1.3939, −1.2608)
Southern Latin America	32.27 (27.40, 38.18)	10.74 (8.10, 14.07)	−2.79 (−3.0158, −2.5552)	426.44 (324.55, 552.22)	384.74 (323.35, 465.73)	−0.10 (−0.1997, 0.0052)
Southern Sub-Saharan Africa	149.51 (115.62, 196.21)	70.87 (55.31, 89.65)	−1.05 (−2.0450, −0.0468)	1204.18 (1012.69, 1409.85)	813.53 (681.66, 958.35)	−1.09 (−1.1450, −1.0294)
Tropical Latin America	93.61 (84.17, 104.29)	20.42 (17.45, 23.48)	−4.06 (−4.4839, −3.6392)	479.50 (385.21, 613.00)	316.27 (270.82, 377.16)	−1.22 (−1.4784, −0.9553)
Western Europe	4.73 (3.72, 6.30)	2.88 (1.82, 4.46)	−1.36 (−1.5111, −1.2036)	190.81 (146.70, 250.20)	208.89 (164.79, 263.71)	0.41 (0.2740, 0.5434)

ASIR, age-standardized incidence rate.

In 2021, the three regions with the highest ASIR and DALYs were Central Sub-Saharan Africa [DALYs: 255.22 (95% UI: 182.06 to 348.26), ASIR: 1323.57 per 100,000 persons (95% UI: 1098.63 to 1609.63)], Eastern Sub-Saharan Africa, and the Caribbean. Conversely, the three regions with the lowest ASIR and DALYs in 2021 were high-income Asia Pacific [DALYs: 1.90 (95% UI: 1.30 to 2.74), ASIR: 154.28 per 100,000 (95% UI: 132.33 to 182.35)], Central Europe, and Western Europe (Table 1; Supplementary Table 4).

The regions that recorded the highest EAPC in DALYs between 1990 and 2021 were the Caribbean [0.75 (95% CI: 0.53 to 0.96)], high-income North America [−0.32 (95% CI: −0.51 to −0.36)], and Oceania. Conversely, the regions with the lowest EAPC in DALYs were East Asia [−5.36 (95% CI: −5.61 to −5.11)], North Africa and the Middle East [−4.78 (95% CI: −4.91 to −4.64)], and Andean Latin America. For ASIR, between 1990 and 2021, the regions with the highest EAPC were Eastern Europe [1.12 (95% CI: 0.65 to 1.59)], Western Europe [0.41 (95% CI: 0.27 to 0.54)], and Central Asia. The regions with the lowest EAPC for ASIR were high-income Asia Pacific [−1.52 (95% CI: −1.99 to −1.05)], South Asia [−2.80 (95% CI: −3.06 to −2.55)], and Southeast Asia (Table 1; Supplementary Table 4).

### 3.2 Global country distribution in the burden of HDP (1990–2021)

The ASIRs per 100,000 population for maternal hypertensive disorders in 2021 ranged widely between countries and regions (Supplementary Table 1). The uppermost ASIR estimates were found in South Sudan [2337.94 (95% UI 2015.37 to 2688.94)], Niger [2175.70 (95% UI 1763.51 to 2598.50)], and Chad [2138.38 (95% UI 1783.73 to 2544.84)] (Supplementary Figure 1 and Supplementary Table 5), whereas the lowest estimates were reported in the Republic of Korea [34.99 (95% UI 28.24 to 42.44)], Canada [39.16 (95% UI 28.94 to 52.54)], and Luxembourg [55.19 (95% UI 43.29 to 70.71)] (Supplementary Table 5).

There was considerable variation in the age-standardized DALY rates across countries in 2021, ranging from 588.43 (95% UI 330.70 to 873.05) in Wajir, 584.62 (95% UI 236.82 to 1150.04) in Gilgit-Baltistan, and 459.99 (95% UI 241.31 to 759.00) in Balochistan to 1.27 (95% UI 0.84 to 1.77) in Austria, 1.18 (95% UI 0.67 to 1.97) in Singapore, and 0.92 (95% UI 0.65 to 1.24) in the Republic of Korea (Supplementary Figure 1 and Supplementary Table 5).

The EAPC in ASIR from 1990 to 2021 varied substantially by country. The Russian Federation [1.53 (95% CI 1.05 to 2.01)], Romania [1.19 (95% CI 0.60 to 1.79)], and Georgia [0.98 (95% CI −1.47 to 3.49)] had the highest increases in ASIR, while Palestine [−3.42 (95% CI −3.79 to −3.04)], Syrian Arab Republic [−2.82 (95% CI −2.87 to −2.76)], and Saint Lucia [−2.62 (95% CI −2.76 to −2.47)] had the highest decreases in ASIR. For DALY rates, the highest EAPC was observed in Guam [1.78 (95% CI 1.32 to 2.24)], American Samoa [0.50 (95% CI 0.11 to 0.89)], and United Republic of Tanzania [−0.11 (95% CI −0.49 to 0.26)], while the lowest EAPC was in Syrian Arab Republic [−7.10 (95% CI −8.01 to −6.18)], Lao People’s Democratic Republic [−6.70 (95% CI −6.87 to −6.52)], and the Republic of Korea [−6.59 (95% CI −6.99 to −6.19)] (Supplementary Figure 1 and Supplementary Table 5).

### 3.3 Age distribution

**Figure 3:**
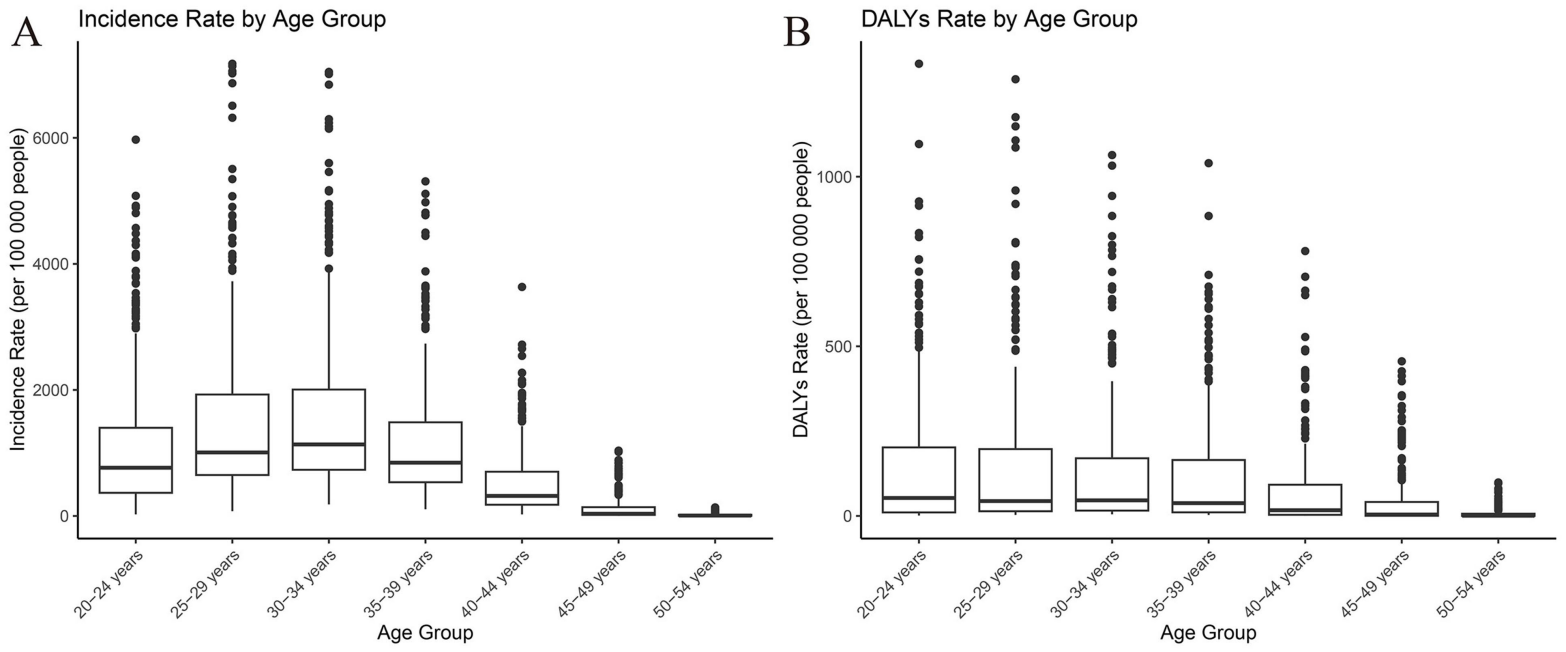
Age distribution of incidence and DALY rates for the global burden of maternal hypertensive disorders in 2021. DALY, disability adjusted life-year.

The incidence and DALY rate of maternal hypertensive disorders were highest among women aged 25–39 years, with a marked decrease in the 50–54-year age group (Figure 3). This finding underscores the significant burden of maternal hypertensive disorders among women of reproductive age, emphasizing the need for tailored healthcare interventions. The heatmap analysis revealed elevated DALY rates in certain regions, particularly among the 25–39 year age group, as indicated by the darker shades (Supplementary Figure 3). Similarly, the ASIR heatmap demonstrates regional disparities, with some areas exhibiting notably higher incidence rates within the same age bracket (Supplementary Figure 4).

There were considerable variations in age-specific DALYs and ASIR for each country; for instance, Afghanistan (25–39 years) and Angola (25–39 years) had the highest rates, while Albania (40–69 years) and Andorra (70–89 years) had the lowest rates. The consistent trend of higher DALYs and ASIR among younger age groups suggests the need for region-specific interventions aimed at decreasing the burden of maternal hypertensive disorders (Supplementary Tables 2, 3). The decline observed in older age groups may reflect age-related effects on the incidence and consequences of maternal hypertensive disorders.

### 3.4 Burden of HDP in regions stratified by SDI

**Figure 4:**
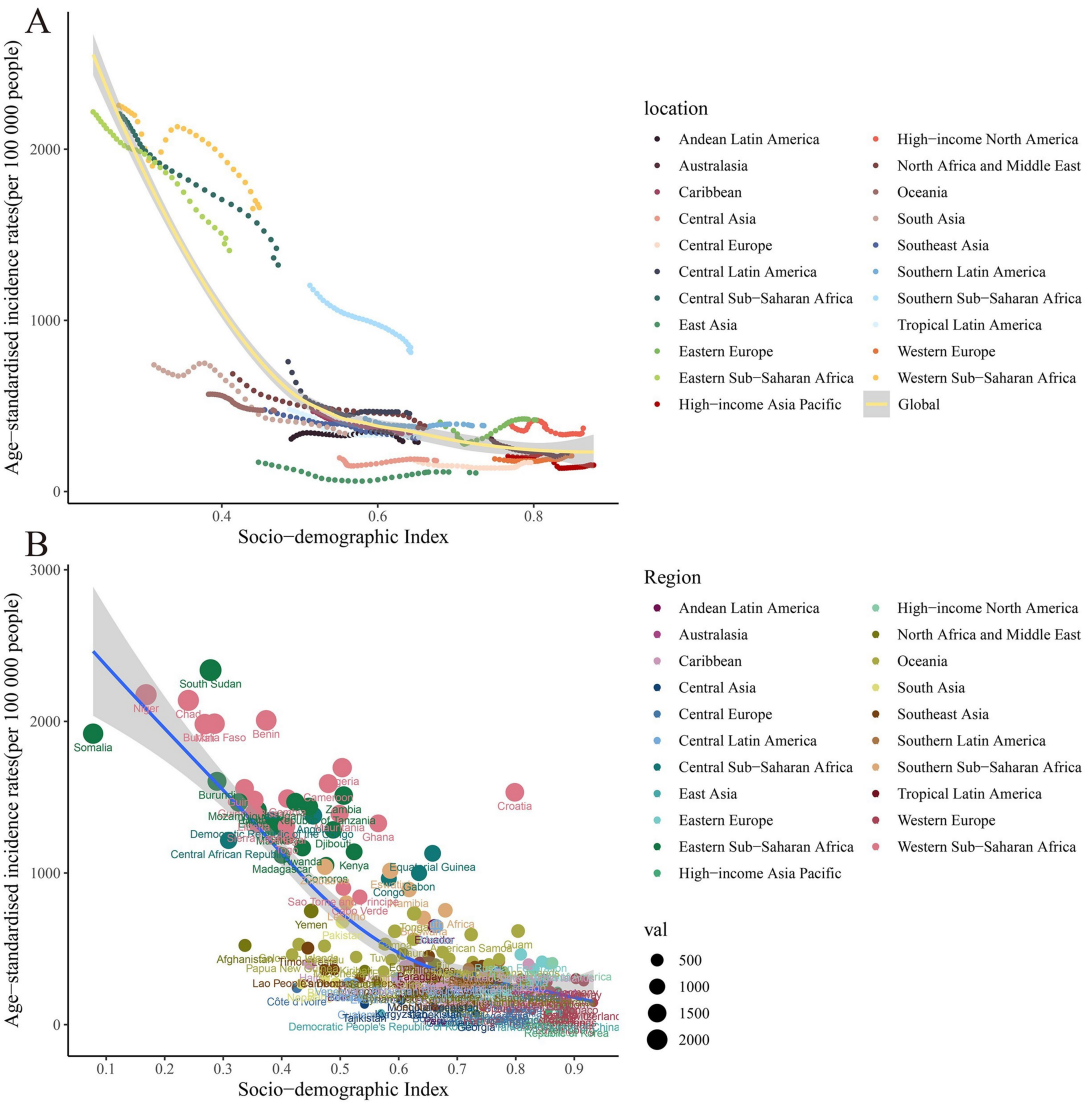
Scatter plot of correlation analysis between ASIR of global burden of maternal hypertensive disorders in 1990 and 2021 and the SDI by locations. ASIR, age-standardized incidence rate; SDI, sociodemographic index.

In 2021, a broad positive correlation was noted between ASIR and SDI across all countries/regions. Those countries/regions with lower SDIs exhibited higher ASIRs (Table 1). The highest incidence rates were observed in Chad, Niger, and Somalia, with corresponding SDIs of 0.24, 0.16, and 0.08, respectively. Conversely, the lowest incidence rates were documented in the Republic of Korea, Canada, and Luxembourg, with their SDIs being 0.88, 0.87, and 0.90, respectively. The surge in global HDP cases can predominantly be attributed to the upward trend in low-SDI regions (Table 1 and Figure 4).

**Figure 5:**
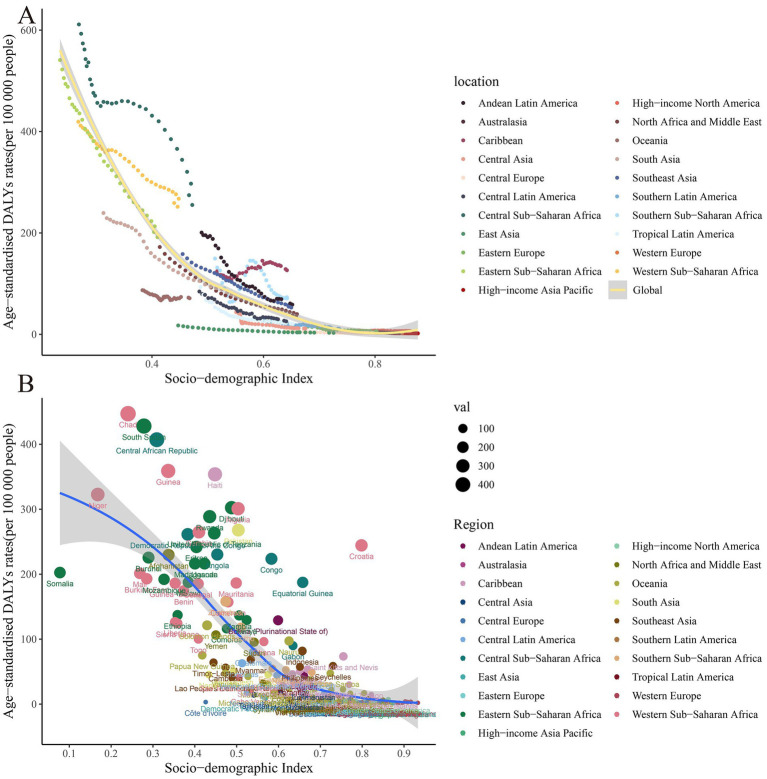
Scatter plot of correlation analysis between age-standardized DALY rates of global burden of maternal hypertensive disorders in 1990 and 2021 and the SDI by location. DALY, disability adjusted life-year.

Similar to the trend observed in age-standardized DALY rates, there was a positive association between SDI and age-standardized DALY rates in 2021. The three countries with the highest DALY rates were Wajir (SDI = 0.35), Gilgit-Baltistan (SDI = 0.42), and Balochistan (SDI = 0.41). These regions with the highest DALY rates were characterized by low SDI values, indicating that maternal hypertensive disorders were more common in less developed areas. On the other hand, the three countries with the lowest DALY rates were the Republic of Korea (SDI = 0.88), Singapore (SDI = 0.87), and Austria (SDI = 0.91). This negative correlation between DALY rates and SDI further reinforces the impact of socio-economic development on health outcomes (Table 1 and Figure 5).

## 4 Discussion

This study presents a comprehensive overview of the global epidemiology of HDP, utilizing DALYs and incidence rates. Both incidence and DALYs have shown a decline over time, attributable to heightened awareness and advancements in medical technology, as previously documented (24, 25). While there was a significant increase in HDP cases during the early 20th century, the rate has since leveled off. However, there has been a continuous increase in the number of cases over the past three decades (26). These insights suggest that the focus should shift toward preventing HDP rather than merely addressing maternal and infant mortality (27). The age-standardized incidence and DALYs have diminished due to enhanced medical care and the global aging population.

The burden of HDP varies widely across regions, with the most significant age-standardized DALYs and ASIR noted in Africa. In sub-Saharan Africa, maternal deaths from HDP constitute approximately 22.1% of all maternal deaths, highlighting the prominence of these conditions in this region (28). This high proportion is largely due to limited access to quality antenatal care and emergency obstetric services, which delays the diagnosis and management of HDP (PMID: 30848083). Additionally, low health literacy and socioeconomic barriers often prevent timely medical intervention (PMID: 32813709). In Central and East Africa, HDP rank as the second most common cause of maternal death, following obstetric hemorrhage (29). The elevated burden correlates with limited healthcare access and reduced rates of early prenatal diagnosis, which presents a significant hurdle to improving maternal and infant outcomes (30). Investments in healthcare have been demonstrated to decrease mortality from HDP (31). In the Caribbean, there has been a steady increase over time in DALY ASR related to HDP, reflecting disparities in primary care and potential environmental exposures, such as chlordecone (32). The persistent increase in DALY rates in the Caribbean reflects regional inequalities in maternal healthcare. In some areas, environmental exposures (e.g., chlordecone contamination), inadequate prenatal care infrastructure, and socioeconomic hardship may contribute to a sustained HDP burden (PMID: 24727072; PMID: 35509645). In contrast, the global trend of declining incidence has not been mirrored in Eastern and Western Europe, where the ASR for HDP has increased. According to the ROPAC survey by the European Society of Cardiology, although adverse outcomes from hemorrhage and infection are less prevalent in Western developed countries, cardiovascular diseases, including HDP, remain a substantial issue (33). According to the ROPAC survey by the European Society of Cardiology, the increasing ASIR in Eastern Europe may be attributed to demographic transitions such as increased maternal age, higher BMI, and multiple pregnancies (PMID: 40311220; PMID: 27384461). In addition, healthcare reforms and limited access to continuous maternal care in countries like Romania have disrupted service quality, potentially leading to higher HDP incidence (PMID: 30907409). These trends may also reflect socioeconomic disparities and gaps in early screening and prevention strategies. Higher maternal BMI, older maternal age, and multiple pregnancies partly account for the increase compared to developing countries (34). Additionally, social determinants like migration patterns and educational attainment levels, as well as genetic susceptibility among different ethnic populations, may also influence regional differences in HDP burden (35).

In regions with lower SDI, several underlying factors contribute to the higher incidence and burden of hypertensive disorders of pregnancy (HDP). These areas commonly experience insufficient medical resources, including limited access to prenatal care and a lack of specialized maternal health services (PMID: 39395960). Additionally, low levels of health literacy and education impede early recognition and management of HDP. Such challenges hinder timely diagnosis, monitoring, and treatment, ultimately increasing adverse maternal outcomes (PMID: 39354425). Conversely, high-SDI regions typically benefit from well-established healthcare infrastructures, comprehensive antenatal care programs, and widespread health education, which collectively reduce the risk and impact of HDP (PMID: 39789321). Thus, the positive correlation between SDI and HDP burden reflects the crucial role of socioeconomic development in shaping maternal health outcomes.

To effectively reduce the burden of HDP in low-SDI regions, targeted interventions are essential. These interventions should include expanding access to quality antenatal care, investing in rural and underserved healthcare infrastructure, training local healthcare providers, and integrating HDP screening into routine maternal health services. Community-based health education programs tailored to cultural and literacy contexts can improve awareness of early HDP symptoms and promote timely medical seeking behavior. Additionally, mobile health platforms may offer scalable solutions to reach remote populations with health education and care reminders, especially in regions with limited facility-based care.

The increase in ASIR in Romania is related to the transition and reform of the healthcare system, which disrupted the continuity and quality of maternal health services (36). The socioeconomic status and the increasing prevalence of obesity and metabolic disorders have also contributed to the increased incidence of HDP (37). The healthcare system has not been able to adapt to these changes, which have affected the management and prevention of hypertensive disorders (38). Recent advances in identifying shared cfDNA methylation profiles among HDP subtypes (39) could offer novel biomarkers for early risk stratification, potentially improving resource allocation in strained healthcare systems. In Georgia, the increase in ASIR is associated with challenges in the healthcare infrastructure, socioeconomic factors, cultural attitudes toward prenatal care, higher smoking rates among pregnant women, and poor nutrition (40). The increase in DALY rates in Guam is due to the high prevalence of obesity and diabetes, limited access to specialized maternal care, cultural dietary practices, and genetic predispositions (41). American Samoa is challenged by a high prevalence of obesity, a lack of adequate healthcare resources, and genetic factors that contribute to the burden of HDP (42). The identification of common epigenetic signatures across the HDP spectrum (39) highlights opportunities for precision public health approaches, particularly in populations with strong genetic predispositions.

Our research further reveals that the burden of HDP is predominantly concentrated among women aged 25 to 39 years. Women in this age group bear the main burden of hypertensive disorders of pregnancy, which may be attributed to several common risk factors. First-time pregnancies (primiparity) are associated with an increased risk of HDP due to the maternal immune system’s adaptation to the fetus (PMID: 29669140). Additionally, women in this age range may experience multiple pregnancies and higher parity, which also elevate the risk of HDP (PMID:34583671). Meanwhile, occupational stress and lifestyle factors prevalent among the working-age population further contribute to the development of HDP (PMID: 34897523). Earlier studies identified HDP as the leading cause of maternal mortality in adolescents, yet it was often overlooked in favor of maternal anemia and preterm delivery (42). The incidence of HDP is notably higher in developing nations and rural regions (32). Adolescent mothers are more prone to engage in detrimental behaviors such as smoking, alcohol consumption, or drug abuse and may have a history of miscarriage, factors that exacerbate the risk of HDP. Typically, these mothers are unmarried, exhibit limited understanding of maternal health, and are less likely to receive consistent prenatal care. These circumstances impede the early diagnosis and treatment of HDP (32). Furthermore, uterine immaturity may also contribute to the incidence of HDP (39). As societal acceptance of sexuality increases, it becomes imperative to bolster educational, medical, and policy efforts aimed at preventing and treating HDP during adolescent pregnancy (43). Beyond behavioral risk factors, pregnant adolescent women also face unique physiological and social vulnerabilities. Immature reproductive systems—such as underdeveloped uterine vasculature—may impair placental development, increasing the risk of HDP (PMID: 35844352). Additionally, adolescents often lack emotional and financial support, which exacerbates stress and limits their ability to access consistent prenatal care (PMID: 17880312). Social stigma, fear of disclosure, and lower educational attainment further compound the barriers to early HDP detection and treatment. These multidimensional risks underscore the importance of adolescent-specific maternal health strategies, including school-based education, confidential antenatal services, and supportive social environments.

In conclusion, our study reveals that HDP remains a significant global public health concern. The epidemiology of HDP exhibits substantial variations across different regions and age groups. Even with the anticipated increase in HDP cases in the coming years, several research challenges persist. Notably, there is an absence of a standard definition or classification for HDP. Additionally, the consistency and quality of data sources and collection methodologies differ considerably, potentially introducing biases in data analysis and comparison. Furthermore, estimates of disease prevalence and incidence should consider the diagnostic capabilities and data collection practices specific to each region.

## 5 Conclusion

Our findings indicate a significant increase in maternal HDP, which is expected to substantially impact the global disease burden. Notably, the ASIR and age-standardized DALY rates remain alarmingly high in Africa and the Caribbean. In the Caribbean, there was a slight uptick in the age-standardized DALY rate, whereas in Eastern and Western Europe, as well as Central Asia, a minor increase in the ASIR was observed. Specific regions such as the Russian Federation, Romania, and Georgia exhibited an upward trajectory in their ASIR. Meanwhile, territories like Guam and American Samoa recorded elevated DALY rates. The most pronounced burden of HDP was noted among women aged 25 to 39 years. Furthermore, adolescent mothers were identified as a vulnerable group, with their susceptibility attributed to unhealthy behaviors and inadequate prenatal care.

## Data Availability

The original contributions presented in the study are included in the article/Supplementary material, further inquiries can be directed to the corresponding author.
