# Supplementary material for: Epidemiological analysis of maternal hypertensive disorders of pregnancy
Source: Front Med (Lausanne). 2025 Aug 1;12:1498694. doi: 10.3389/fmed.2025.1498694 (PMC12354582; doi:10.3389/fmed.2025.1498694)

**SFigure 1: ASRs and EAPC of global burden of Age-standardized DALY rate of maternal hypertensive disorders in 204 countries in 1990 and 2021, by locations.** DALY = disability adjusted life-year.

**
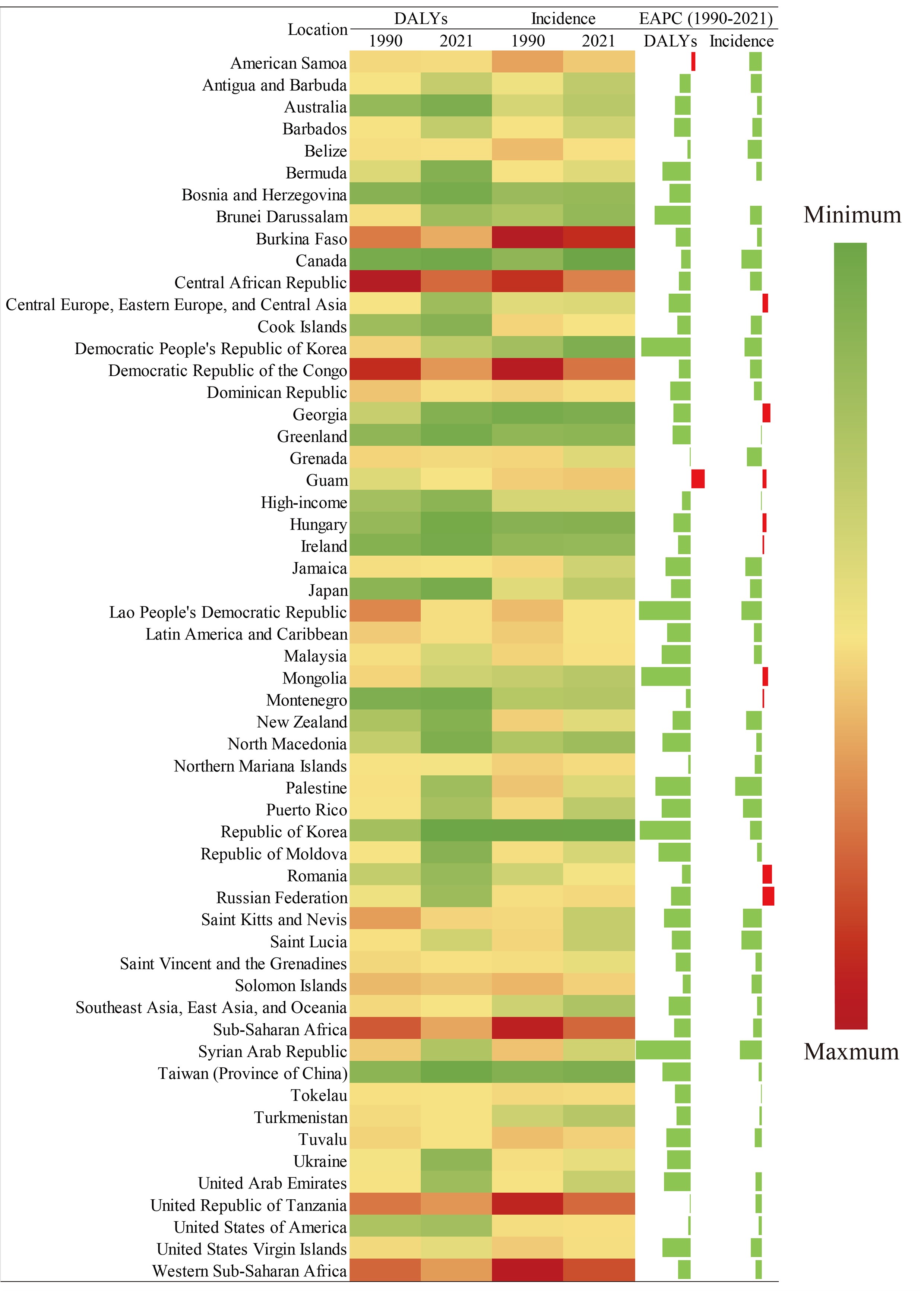
**

**SFigure 2: Trend of global burden of ASIR and Age-standardized DALY rate of maternal hypertensive disorders in regions from 1990 to 2021.** DALY = disability adjusted life-year. ASIR= Age-standardized incidence rate

**
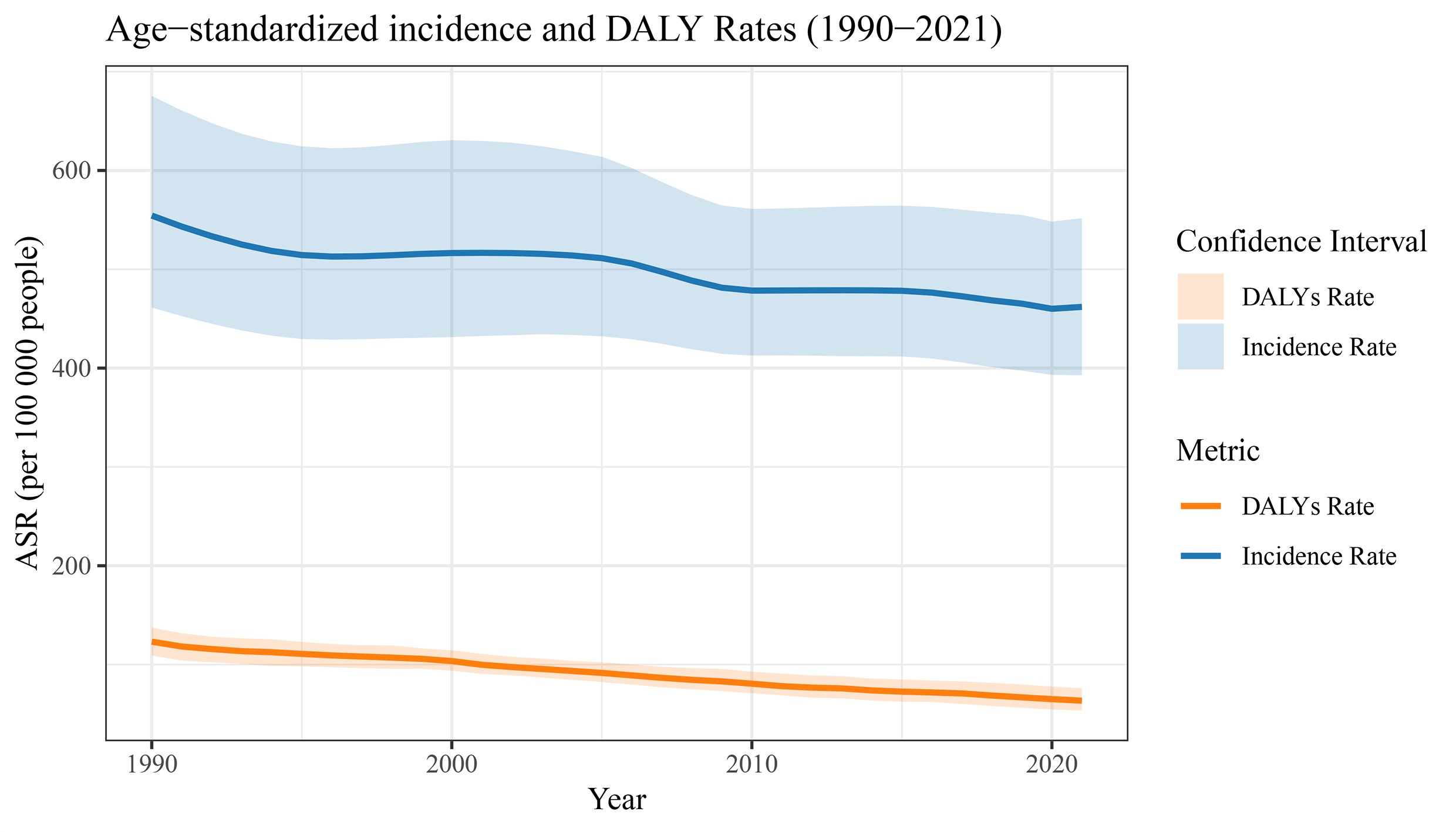
**

**SFigure 3: Global age distribution of Age-standardized DALY rate of maternal hypertensive disorders in different regions in 2021.** DALY = disability adjusted life-year.


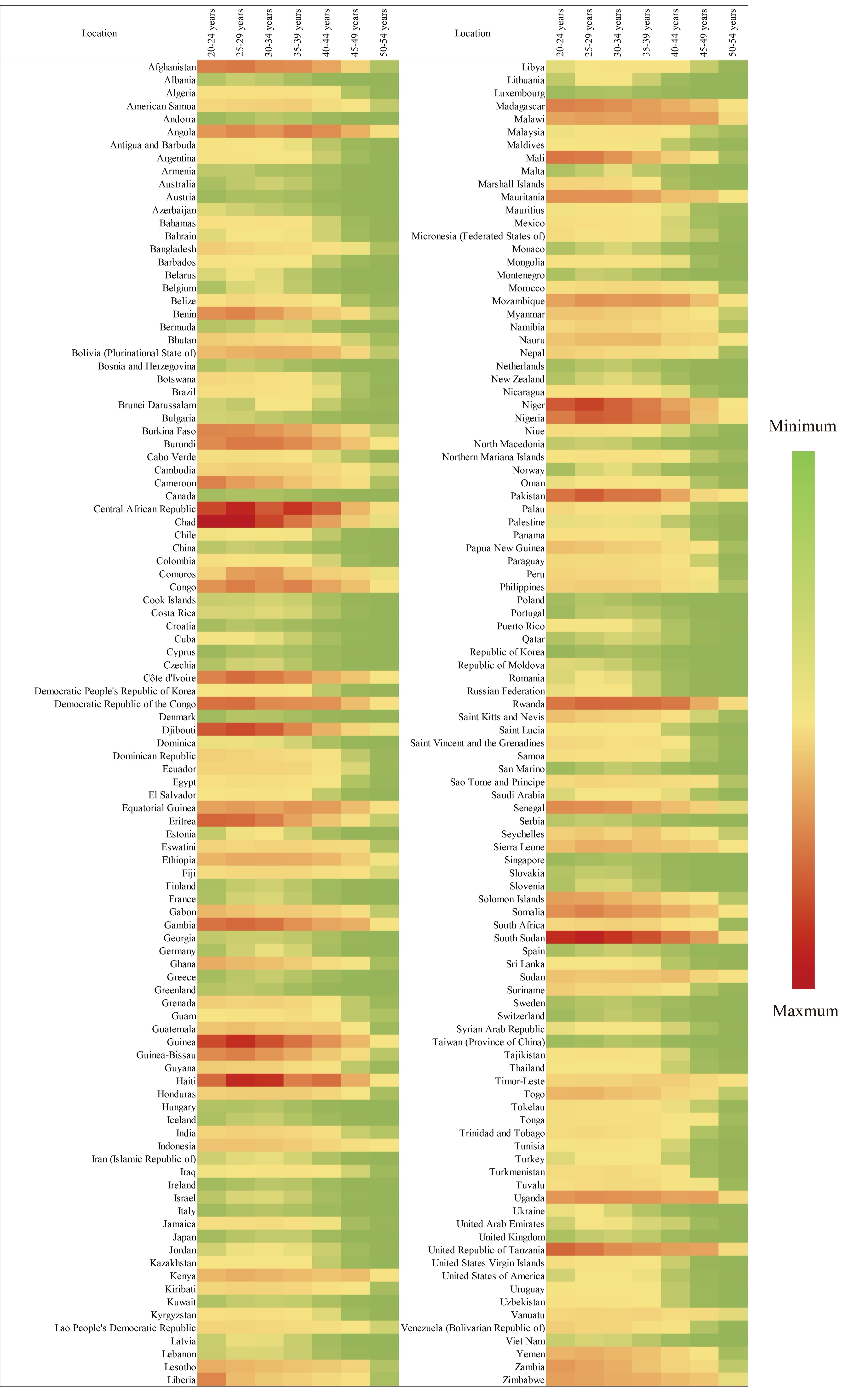


**SFigure 4: Global age distribution of ASIR of maternal hypertensive disorders in different regions in 2021.** ASIR= Age-standardized incidence rate


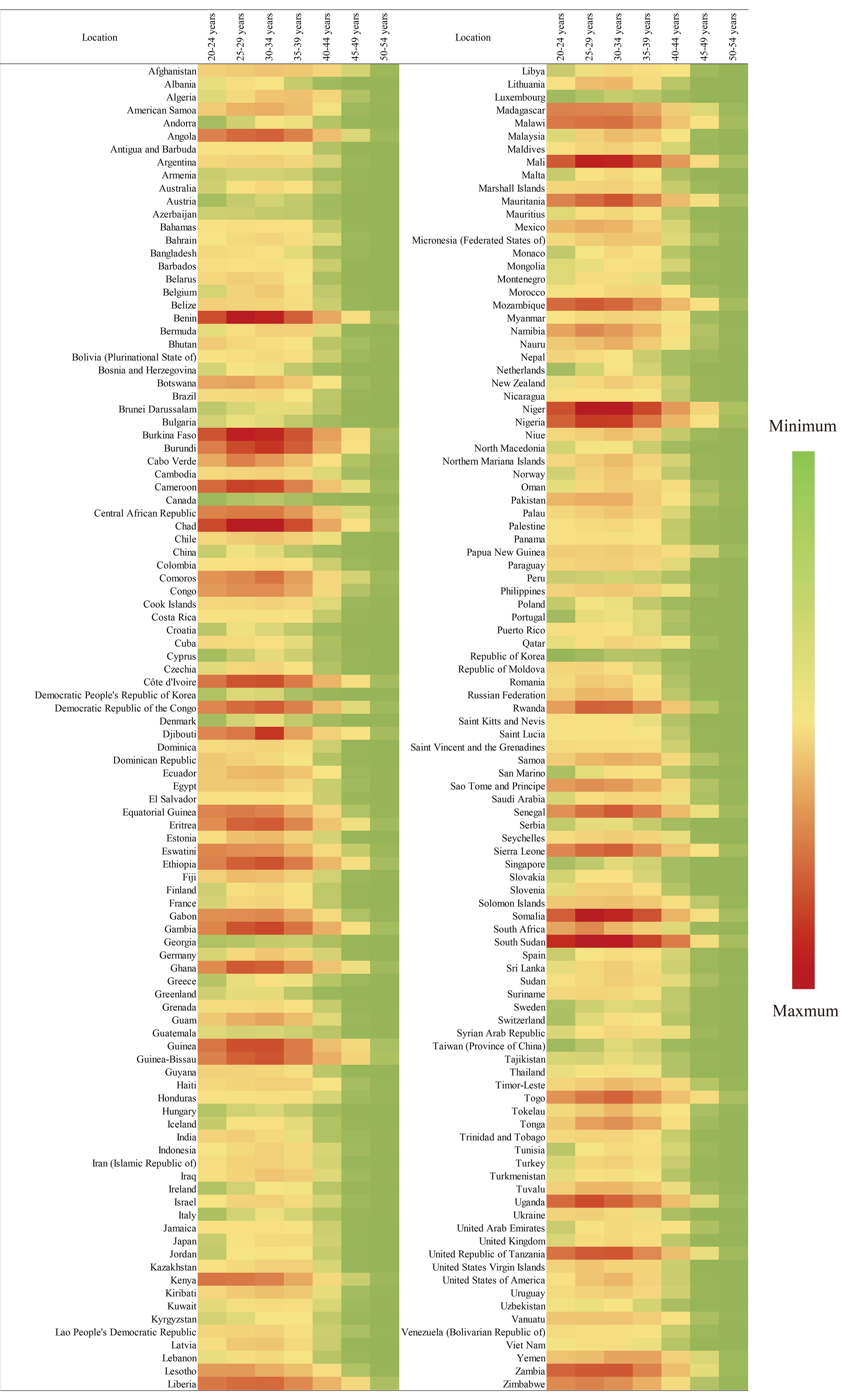

Supplement: Supplementary file 1 [file Data_Sheet_1.docx]
