# Supplementary material for: Epidemiological analysis of maternal hypertensive disorders of pregnancy
Source: Front Med (Lausanne). 2025 Aug 1;12:1498694. doi: 10.3389/fmed.2025.1498694 (PMC12354582; doi:10.3389/fmed.2025.1498694)
Supplement: Supplementary file 2 [file Data_Sheet_2.docx]

STable 1. DALYs and Incidence of maternal hypertensive disorders in 1990 and 2021 for female and all nations.

| location | DALYs (Disability-Adjusted Life Years) | | Incidence | |
| --- | --- | --- | --- | --- |
|  | 1990 | 2021 | 1990 | 2021 |
| location | DALYs (Disability-Adjusted Life Years) _1990 | DALYs (Disability-Adjusted Life Years) _2021 | Incidence_1990 | Incidence_2021 |
| American Samoa | 56.58 (35.17, 81.80) | 47.52 (29.49, 72.12) | 925.65 (729.16, 1195.37) | 594.34 (467.45, 759.45) |
| Antigua and Barbuda | 20.33 (16.74, 24.40) | 10.90 (9.06, 13.35) | 327.47 (253.60, 418.69) | 223.40 (176.21, 281.57) |
| Australia | 5.54 (3.96, 7.58) | 2.81 (1.69, 4.51) | 274.30 (231.50, 324.70) | 209.91 (155.62, 271.88) |
| Barbados | 23.89 (19.50, 28.82) | 10.63 (8.23, 14.25) | 367.06 (289.09, 486.51) | 257.92 (205.09, 334.39) |
| Belize | 42.64 (35.09, 52.63) | 29.34 (23.74, 36.01) | 721.42 (559.32, 926.11) | 386.26 (306.31, 501.67) |
| Bermuda | 13.53 (10.83, 17.19) | 3.60 (2.24, 5.87) | 363.49 (282.35, 477.78) | 292.08 (234.63, 371.96) |
| Bosnia and Herzegovina | 4.07 (2.86, 5.67) | 2.29 (1.41, 3.44) | 139.38 (101.61, 188.33) | 131.28 (95.64, 169.84) |
| Brunei Darussalam | 36.18 (21.59, 56.61) | 6.61 (4.22, 9.95) | 185.48 (145.53, 245.39) | 124.47 (96.99, 157.88) |
| Burkina Faso | 355.55 (253.73, 473.88) | 192.96 (129.41, 273.99) | 2361.32 (1997.31, 2781.91) | 1984.45 (1681.19, 2324.57) |
| Canada | 2.31 (1.63, 3.13) | 1.48 (1.14, 1.95) | 114.64 (95.73, 134.77) | 39.16 (28.94, 52.54) |
| Central African Republic | 736.27 (517.66, 952.94) | 407.03 (253.60, 622.85) | 1948.42 (1598.80, 2369.93) | 1214.92 (977.12, 1503.69) |
| Central Europe, Eastern Europe, and Central Asia | 17.77 (15.46, 20.76) | 6.60 (4.96, 9.04) | 299.05 (228.03, 404.97) | 287.17 (227.74, 363.68) |
| Cook Islands | 6.54 (3.77, 10.75) | 4.01 (2.17, 6.77) | 492.62 (374.88, 650.33) | 348.85 (274.73, 447.38) |
| Democratic People's Republic of Korea | 78.42 (41.82, 127.80) | 9.97 (5.59, 16.97) | 158.61 (116.98, 218.32) | 77.15 (56.98, 103.75) |
| Democratic Republic of the Congo | 605.18 (401.56, 841.42) | 261.31 (172.80, 362.75) | 2284.56 (1920.52, 2666.26) | 1347.68 (1108.49, 1641.78) |
| Dominican Republic | 124.39 (97.40, 152.15) | 42.83 (32.02, 56.56) | 519.26 (410.28, 672.93) | 400.48 (315.19, 524.71) |
| Georgia | 11.13 (8.44, 14.93) | 3.55 (2.82, 4.36) | 63.78 (51.43, 79.20) | 74.59 (70.32, 78.63) |
| Greenland | 5.03 (3.26, 7.19) | 2.20 (1.42, 3.16) | 115.81 (86.56, 154.66) | 111.04 (83.13, 151.51) |
| Grenada | 71.38 (58.20, 86.25) | 53.68 (41.59, 67.65) | 486.43 (381.08, 631.20) | 289.49 (225.87, 366.14) |
| Guam | 13.65 (9.19, 19.30) | 16.47 (11.56, 22.65) | 560.92 (430.18, 720.40) | 617.40 (482.97, 795.03) |
| High-income | 7.31 (5.95, 9.31) | 4.67 (3.31, 6.49) | 272.09 (214.01, 356.03) | 273.31 (230.06, 327.91) |
| Hungary | 5.60 (4.65, 6.80) | 1.93 (1.36, 2.63) | 96.92 (71.91, 133.42) | 95.94 (74.11, 123.86) |
| Ireland | 3.77 (2.88, 4.93) | 1.94 (1.26, 2.95) | 121.85 (90.22, 162.59) | 128.13 (95.89, 167.83) |
| Jamaica | 39.52 (31.86, 48.66) | 25.26 (17.52, 36.42) | 466.62 (364.46, 619.20) | 257.32 (205.98, 325.01) |
| Japan | 4.64 (3.27, 6.85) | 2.40 (1.57, 3.64) | 295.71 (230.45, 389.19) | 217.56 (188.08, 257.15) |
| Lao People's Democratic Republic | 312.60 (212.34, 441.60) | 41.15 (21.64, 73.32) | 720.39 (561.98, 895.50) | 362.64 (286.04, 453.81) |
| Latin America and Caribbean | 102.96 (95.99, 110.55) | 34.99 (29.70, 41.42) | 577.69 (482.68, 705.63) | 375.24 (328.67, 439.97) |
| Malaysia | 40.62 (26.54, 56.89) | 13.06 (9.70, 17.23) | 506.62 (388.93, 665.75) | 388.37 (295.71, 500.51) |
| Mongolia | 70.66 (47.82, 96.60) | 11.85 (8.17, 16.82) | 233.98 (180.31, 298.65) | 204.23 (158.28, 266.04) |
| Montenegro | 3.09 (1.84, 4.85) | 2.47 (1.35, 4.18) | 200.29 (148.81, 269.04) | 199.21 (143.81, 264.91) |
| New Zealand | 8.27 (5.27, 12.63) | 3.74 (2.28, 5.90) | 544.81 (411.71, 706.84) | 297.94 (251.81, 358.49) |
| North Macedonia | 10.76 (7.47, 14.43) | 3.11 (2.00, 4.72) | 186.46 (139.75, 256.05) | 146.08 (105.76, 192.81) |
| Northern Mariana Islands | 24.18 (14.24, 37.14) | 16.46 (10.63, 24.01) | 529.68 (407.87, 697.37) | 431.81 (339.65, 562.05) |
| Palestine | 32.44 (19.49, 48.48) | 6.88 (4.56, 9.84) | 643.68 (496.83, 826.80) | 286.34 (219.35, 371.15) |
| Puerto Rico | 23.86 (19.69, 29.14) | 7.68 (5.82, 10.24) | 456.78 (352.75, 619.55) | 218.21 (170.62, 284.58) |
| Republic of Korea | 7.28 (5.64, 9.05) | 0.92 (0.65, 1.24) | 37.75 (30.11, 47.37) | 34.99 (28.24, 42.44) |
| Republic of Moldova | 16.60 (13.28, 20.81) | 4.03 (2.45, 6.54) | 401.35 (292.16, 568.02) | 275.36 (205.55, 370.46) |
| Romania | 10.81 (8.53, 13.90) | 5.78 (3.87, 8.81) | 257.33 (185.93, 351.58) | 341.25 (256.43, 468.19) |
| Russian Federation | 15.52 (12.40, 19.59) | 6.37 (3.87, 10.07) | 409.78 (299.27, 561.08) | 463.10 (362.33, 588.84) |
| Saint Kitts and Nevis | 242.94 (207.64, 283.52) | 73.48 (56.48, 93.67) | 456.65 (354.70, 588.36) | 233.22 (185.15, 304.15) |
| Saint Lucia | 33.29 (27.43, 40.18) | 12.17 (9.55, 15.65) | 487.34 (377.80, 619.26) | 233.44 (186.76, 297.20) |
| Saint Vincent and the Grenadines | 62.25 (51.02, 75.89) | 32.51 (26.00, 40.94) | 427.58 (329.69, 542.45) | 314.04 (246.84, 402.30) |
| Solomon Islands | 157.32 (72.49, 255.97) | 121.33 (77.14, 178.99) | 760.36 (588.61, 962.22) | 529.54 (412.09, 679.70) |
| Southeast Asia, East Asia, and Oceania | 55.22 (46.29, 64.49) | 21.93 (18.21, 26.94) | 253.38 (198.36, 330.84) | 183.35 (147.40, 230.78) |
| Sub-Saharan Africa | 455.05 (384.37, 528.16) | 214.79 (176.41, 262.04) | 2109.54 (1793.41, 2444.04) | 1461.71 (1252.39, 1693.56) |
| Syrian Arab Republic | 101.75 (66.07, 146.72) | 8.66 (5.31, 13.28) | 656.60 (497.95, 847.03) | 259.93 (196.68, 338.25) |
| Taiwan (Province of China) | 4.40 (3.59, 5.48) | 1.46 (1.00, 2.13) | 89.11 (66.47, 120.32) | 74.85 (55.13, 101.14) |
| Tokelau | 34.20 (18.83, 58.52) | 23.17 (15.05, 35.12) | 452.72 (340.40, 576.43) | 436.31 (331.97, 561.28) |
| Turkmenistan | 52.67 (41.67, 66.86) | 27.32 (19.56, 39.33) | 249.92 (193.59, 326.71) | 203.08 (156.13, 266.53) |
| Tuvalu | 74.26 (40.62, 139.41) | 28.56 (16.94, 45.54) | 683.80 (518.63, 888.00) | 530.91 (409.41, 674.89) |
| Ukraine | 16.30 (13.37, 20.32) | 5.02 (3.06, 7.79) | 414.85 (298.84, 570.58) | 310.19 (235.12, 409.27) |
| United Arab Emirates | 24.51 (13.58, 40.39) | 6.49 (4.25, 9.45) | 367.14 (325.28, 418.30) | 237.51 (183.36, 303.55) |
| United Republic of Tanzania | 363.86 (282.09, 454.32) | 263.17 (179.17, 365.90) | 2063.87 (1720.68, 2401.99) | 1436.86 (1210.42, 1664.67) |
| United States of America | 8.29 (6.09, 11.71) | 7.13 (5.09, 9.61) | 422.36 (322.24, 565.35) | 406.27 (349.93, 476.00) |
| United States Virgin Islands | 54.51 (32.85, 80.81) | 14.30 (9.69, 20.78) | 588.20 (459.23, 787.48) | 398.19 (315.35, 511.46) |
| Western Sub-Saharan Africa | 419.31 (329.99, 508.31) | 251.68 (196.22, 325.42) | 2256.21 (1933.87, 2588.36) | 1678.15 (1447.02, 1911.36) |

**STable 2. Age distribution of Incidence for maternal hypertensive disorders in different countries in 2021.**

| **location** | **sex** | **20-24 years** | **25-29 years** | **30-34 years** | **35-39 years** | **40-44 years** | **45-49 years** | **50-54 years** |
| --- | --- | --- | --- | --- | --- | --- | --- | --- |
| Afghanistan | Female | 1127.652777 | 1318.771376 | 1479.615002 | 1398.697916 | 1017.536633 | 347.7421574 | 41.14164448 |
| Albania | Female | 453.7363429 | 714.2905287 | 562.96468 | 260.9628564 | 65.40695688 | 7.2264757 | 1.038771523 |
| Algeria | Female | 399.4914395 | 914.8445494 | 1466.651081 | 1576.101034 | 985.9109789 | 143.8958347 | 17.50136696 |
| American Samoa | Female | 1335.173501 | 1988.294042 | 2122.003614 | 1691.869721 | 495.8838137 | 42.01568961 | 7.365376054 |
| Andorra | Female | 99.28401971 | 310.6011926 | 595.6813624 | 486.6751187 | 170.5272039 | 13.80849007 | 1.973736144 |
| Angola | Female | 3416.190984 | 4159.85565 | 4339.900626 | 3410.608225 | 1684.782294 | 384.6752847 | 51.0888706 |
| Antigua and Barbuda | Female | 625.7967665 | 652.4801869 | 686.9700984 | 519.0105616 | 160.4577658 | 9.759864068 | 2.041169564 |
| Argentina | Female | 935.9723396 | 1102.180695 | 1179.943659 | 969.2145149 | 357.6004337 | 35.38861155 | 4.475558869 |
| Armenia | Female | 276.5221228 | 329.2557163 | 328.3006279 | 293.2293834 | 88.15703955 | 12.72827248 | 1.640278175 |
| Australia | Female | 308.460826 | 658.8713565 | 934.8005174 | 678.1923734 | 219.7209494 | 21.11946854 | 2.485131099 |
| Austria | Female | 94.09117627 | 257.9069632 | 345.1804059 | 247.5942273 | 66.8428095 | 11.31439309 | 1.68532767 |
| Azerbaijan | Female | 297.1964066 | 293.6524899 | 246.0981018 | 225.6710523 | 80.81709206 | 8.525979633 | 1.224965195 |
| Bahamas | Female | 590.9636416 | 720.5021543 | 734.0164472 | 682.0241206 | 249.0738735 | 18.70358235 | 3.435340133 |
| Bahrain | Female | 546.9033259 | 880.4797881 | 1051.031727 | 784.3868671 | 399.5982711 | 40.21425583 | 5.627137047 |
| Bangladesh | Female | 915.6996388 | 838.4427122 | 668.5941098 | 416.9208511 | 124.5818708 | 35.07848474 | 4.532766467 |
| Barbados | Female | 698.9292771 | 683.0294499 | 758.2291699 | 596.9240628 | 281.7906659 | 10.84922665 | 2.438290392 |
| Belarus | Female | 930.8685758 | 1254.689807 | 1051.899177 | 534.9457773 | 121.4710056 | 6.332372526 | 0.817064613 |
| Belgium | Female | 352.5227569 | 1119.907676 | 1366.420004 | 713.2418267 | 223.0366629 | 19.85963932 | 2.634446213 |
| Belize | Female | 1165.679403 | 1107.818 | 1009.93689 | 801.3027728 | 282.3986656 | 27.74881194 | 5.41757795 |
| Benin | Female | 4922.440018 | 7022.835515 | 6296.526139 | 4443.768814 | 2268.715502 | 754.742613 | 103.9143628 |
| Bermuda | Female | 429.5452574 | 703.8699624 | 1200.995239 | 1139.267066 | 414.3045903 | 30.85155921 | 5.521598569 |
| Bhutan | Female | 1320.144033 | 936.7215251 | 789.6672235 | 535.1143286 | 193.7368783 | 95.01030974 | 11.83118602 |
| Bolivia (Plurinational State of) | Female | 607.9740799 | 709.8276487 | 872.967969 | 775.2400002 | 277.4484763 | 49.93969661 | 8.733659731 |
| Bosnia and Herzegovina | Female | 335.7334317 | 559.105066 | 489.067968 | 240.6888135 | 62.68687634 | 5.037210175 | 0.723336218 |
| Botswana | Female | 2348.861457 | 2492.158579 | 1973.154137 | 1416.197883 | 561.6327433 | 58.52441413 | 10.54400218 |
| Brazil | Female | 850.0086466 | 900.5999798 | 1002.856647 | 568.0987973 | 209.4326025 | 25.03949 | 5.350853204 |
| Brunei Darussalam | Female | 222.4684709 | 362.1839891 | 438.7801779 | 438.4939026 | 205.9833355 | 10.12321382 | 1.594966897 |
| Bulgaria | Female | 323.4374741 | 461.7785268 | 408.1273898 | 231.7917197 | 79.91745377 | 15.20284391 | 2.141279304 |
| Burkina Faso | Female | 4805.223522 | 6509.864038 | 6238.644344 | 4774.791509 | 2540.185581 | 840.3867125 | 110.2037494 |
| Burundi | Female | 3536.580496 | 5071.747489 | 5599.937163 | 4496.936767 | 2153.130304 | 654.8075382 | 83.00005784 |
| Cabo Verde | Female | 2238.952921 | 3478.802414 | 2708.461443 | 1708.996621 | 683.4332378 | 154.7037355 | 23.61244778 |
| Cambodia | Female | 909.2175289 | 1082.83176 | 1166.174721 | 907.7337447 | 388.0200226 | 95.44416498 | 13.14960708 |
| Cameroon | Female | 4161.440166 | 5345.168289 | 5150.585531 | 3416.500266 | 1549.92677 | 430.0843725 | 57.91814291 |
| Canada | Female | 50.94734223 | 144.1981459 | 196.1730876 | 105.871957 | 26.69651778 | 2.758930539 | 0.533701401 |
| Central African Republic | Female | 3375.967025 | 3617.09621 | 3623.025824 | 2735.080535 | 1416.372468 | 400.6444976 | 52.51828648 |
| Chad | Female | 5079.884419 | 7058.079816 | 7046.72106 | 4977.565616 | 2272.931308 | 668.4291026 | 86.95375598 |
| Chile | Female | 880.8702344 | 1298.413899 | 1520.755055 | 1189.448621 | 475.96201 | 29.73420623 | 3.690040604 |
| China | Female | 268.0845183 | 484.1209183 | 414.6109234 | 209.3887313 | 68.61968061 | 14.07339295 | 2.880287527 |
| Colombia | Female | 591.5249749 | 671.1807042 | 738.8587621 | 650.8157142 | 289.8035261 | 40.51142296 | 10.74168731 |
| Comoros | Female | 2898.219001 | 3202.740496 | 3862.080951 | 2543.508835 | 962.3282452 | 335.2377136 | 44.54993134 |
| Congo | Female | 2722.80388 | 3061.614661 | 3100.160993 | 2272.901258 | 881.0722134 | 159.9256196 | 22.66213605 |
| Cook Islands | Female | 928.379492 | 997.6396652 | 1133.038948 | 966.3571015 | 399.2689313 | 36.87617402 | 5.944918212 |
| Costa Rica | Female | 621.9366897 | 655.7027635 | 726.237495 | 617.1134885 | 248.8652419 | 20.19611655 | 6.265693494 |
| Croatia | Female | 206.6299799 | 480.0459681 | 384.3834993 | 222.9605607 | 56.07658171 | 8.376165485 | 1.343396789 |
| Cuba | Female | 907.5949301 | 858.5434391 | 693.731085 | 434.4191133 | 124.5036866 | 7.570960443 | 1.816585223 |
| Cyprus | Female | 92.2757932 | 263.8371863 | 432.5003031 | 301.7251949 | 109.8811065 | 22.3594451 | 3.941567844 |
| Czechia | Female | 416.0947661 | 926.2647591 | 1085.131782 | 603.9648909 | 162.2767456 | 12.2709858 | 1.627742568 |
| Côte d'Ivoire | Female | 3797.760164 | 4760.25796 | 4883.062466 | 3613.438629 | 1938.708317 | 714.1615809 | 91.79738334 |
| Democratic People's Republic of Korea | Female | 144.2015192 | 385.0872654 | 360.2014441 | 113.5811657 | 24.48653173 | 4.149651343 | 0.960471571 |
| Democratic Republic of the Congo | Female | 3241.928164 | 4114.407018 | 4517.728941 | 3487.080608 | 1695.047787 | 410.0483247 | 54.0654886 |
| Denmark | Female | 92.38159626 | 342.4027999 | 439.6977822 | 245.8589474 | 77.85750217 | 8.358125837 | 2.124625689 |
| Djibouti | Female | 3215.043555 | 3703.419875 | 5599.578665 | 2488.850428 | 1137.413995 | 638.5328401 | 81.02857898 |
| Dominica | Female | 815.5802679 | 953.2041746 | 1033.748071 | 802.6628503 | 298.9594849 | 18.14996778 | 3.915052829 |
| Dominican Republic | Female | 1382.338258 | 1123.413589 | 922.8081793 | 560.907172 | 164.8393408 | 21.84285858 | 4.145290926 |
| Ecuador | Female | 1360.638031 | 1788.857404 | 1902.718482 | 1464.181156 | 544.3788134 | 41.61985036 | 7.615867875 |
| Egypt | Female | 1398.471247 | 1353.867327 | 1456.955823 | 917.9147032 | 280.0259677 | 67.71930423 | 9.24038279 |
| El Salvador | Female | 613.3362796 | 628.1984867 | 683.9868686 | 566.1690997 | 284.2983196 | 24.36369286 | 6.802891274 |
| Equatorial Guinea | Female | 3346.092594 | 3602.368388 | 3382.081775 | 2080.733977 | 961.9708309 | 149.0013531 | 22.01335666 |
| Eritrea | Female | 3046.149832 | 4325.388037 | 4569.322594 | 3278.343831 | 1498.064813 | 481.3153499 | 61.60603299 |
| Estonia | Female | 700.6812627 | 1601.927843 | 1792.007108 | 1075.459684 | 338.0657818 | 17.68881691 | 2.193185942 |
| Eswatini | Female | 3265.166157 | 3022.870442 | 2947.421507 | 1991.909027 | 830.6755441 | 254.2050923 | 36.71431822 |
| Ethiopia | Female | 3414.693353 | 4414.468211 | 4822.661691 | 3642.302512 | 1866.673849 | 714.3608925 | 98.0846491 |
| Fiji | Female | 1212.124159 | 1748.596101 | 1685.519049 | 1168.876148 | 322.554493 | 35.93395059 | 5.858033015 |
| Finland | Female | 302.6796148 | 730.0922072 | 951.6935781 | 614.6033257 | 211.5429746 | 17.38804474 | 2.360026514 |
| France | Female | 338.0215095 | 940.4218058 | 1106.73247 | 674.1059943 | 224.6248286 | 19.59715876 | 2.642451022 |
| Gabon | Female | 2977.029555 | 3080.492159 | 3208.668307 | 2259.23818 | 866.0583411 | 136.0803768 | 19.78375425 |
| Gambia | Female | 3284.030589 | 4772.945668 | 5168.978125 | 3880.806779 | 2090.108439 | 666.0326634 | 88.45715385 |
| Georgia | Female | 135.5109743 | 173.1961096 | 257.0415339 | 275.4324441 | 120.7464275 | 16.09284558 | 2.266635276 |
| Germany | Female | 359.1562453 | 940.1988343 | 1435.579142 | 1035.389855 | 337.7717004 | 24.31172416 | 4.204734448 |
| Ghana | Female | 3136.59571 | 4626.7621 | 4429.297715 | 3169.105738 | 1507.248981 | 488.0450229 | 65.07473494 |
| Greece | Female | 180.3511904 | 446.285643 | 675.2473936 | 477.4643896 | 176.0925721 | 40.96502553 | 5.366719712 |
| Greenland | Female | 312.6039739 | 436.305168 | 417.0097389 | 198.8845417 | 37.15102859 | 4.16394204 | 0.854234235 |
| Grenada | Female | 784.0508166 | 827.8111651 | 934.8067524 | 690.5909603 | 261.2876051 | 17.57000539 | 3.432093302 |
| Guam | Female | 1383.97063 | 2057.871493 | 2374.785129 | 1712.047483 | 408.2206028 | 34.86731428 | 6.147949838 |
| Guatemala | Female | 392.5748752 | 343.2558044 | 367.7745611 | 305.3353036 | 192.3959045 | 30.12561725 | 10.35240505 |
| Guinea | Female | 3785.714408 | 4905.108439 | 4949.441864 | 3629.674451 | 1667.578529 | 880.6646191 | 115.0992182 |
| Guinea-Bissau | Female | 3462.004249 | 4409.55447 | 4792.034073 | 3608.998491 | 2110.182226 | 1017.082624 | 132.5501019 |
| Guyana | Female | 1080.790997 | 1071.961703 | 1007.667211 | 739.8435374 | 207.5239437 | 25.97584775 | 4.676038168 |
| Haiti | Female | 898.5088478 | 1021.915289 | 1221.19539 | 1181.862648 | 545.0626845 | 100.432803 | 14.34282522 |
| Honduras | Female | 650.3855811 | 669.3514652 | 742.690959 | 702.5052814 | 366.4883947 | 64.50722659 | 16.89421234 |
| Hungary | Female | 173.8388883 | 319.9087364 | 381.9036968 | 248.9898712 | 85.46575248 | 7.128126091 | 1.045500347 |
| Iceland | Female | 255.1062643 | 597.4636078 | 648.5220105 | 428.2178878 | 146.5077666 | 17.69888332 | 3.136824129 |
| India | Female | 1019.310529 | 1252.474696 | 911.6645945 | 448.7336758 | 153.0884662 | 51.87223297 | 6.534638945 |
| Indonesia | Female | 538.5224783 | 894.983998 | 1166.784203 | 910.4986384 | 355.7565985 | 71.35779903 | 10.30087647 |
| Iran (Islamic Republic of) | Female | 702.0004889 | 1059.742203 | 1158.380549 | 867.8590682 | 332.7296261 | 26.69645746 | 3.626434009 |
| Iraq | Female | 742.7603566 | 1035.001684 | 1478.319356 | 1269.032143 | 394.7978986 | 72.42153122 | 9.957363765 |
| Ireland | Female | 151.6999666 | 321.3432086 | 567.4836274 | 506.2459007 | 191.0600812 | 23.54128051 | 4.677120992 |
| Israel | Female | 570.3068655 | 1082.461449 | 1177.704577 | 853.6844916 | 332.5879002 | 56.13340731 | 7.044035983 |
| Italy | Female | 125.9769705 | 329.9988194 | 481.6276904 | 351.938616 | 155.8138907 | 30.41193441 | 5.966456468 |
| Jamaica | Female | 673.9840622 | 651.1228868 | 728.2706816 | 612.5881424 | 294.4647652 | 21.05965944 | 3.942642069 |
| Japan | Female | 265.6592917 | 598.3593701 | 889.6828109 | 908.0121901 | 316.8707323 | 12.1049623 | 2.221240805 |
| Jordan | Female | 252.2803442 | 527.5086431 | 623.0047875 | 528.9205808 | 260.1601714 | 20.57527653 | 3.830316844 |
| Kazakhstan | Female | 626.9961588 | 868.7859881 | 1219.439504 | 1181.574241 | 367.2463544 | 21.32750429 | 2.817640882 |
| Kenya | Female | 3805.36107 | 3694.1561 | 3418.426067 | 2247.682997 | 890.9756035 | 275.8731216 | 37.37202879 |
| Kiribati | Female | 968.7698101 | 1350.509288 | 1629.679185 | 1389.606871 | 437.8566112 | 82.35567686 | 11.89330737 |
| Kuwait | Female | 427.2579919 | 719.3946458 | 838.3531295 | 762.5793968 | 364.54645 | 56.90022401 | 7.581772054 |
| Kyrgyzstan | Female | 338.4882125 | 409.8740046 | 535.7385017 | 554.2899408 | 241.2153784 | 34.9160581 | 4.214923501 |
| Lao People's Democratic Republic | Female | 1002.807791 | 1015.697136 | 1133.41824 | 771.5174017 | 291.8053689 | 116.1283131 | 15.83848381 |
| Latvia | Female | 768.6894657 | 1389.960297 | 1451.171926 | 910.6032811 | 273.2865429 | 16.93189189 | 2.11226995 |
| Lebanon | Female | 444.3750692 | 783.1702543 | 870.8605745 | 549.6965733 | 189.7275758 | 34.74368797 | 4.946382269 |
| Lesotho | Female | 2662.085292 | 2729.751806 | 2114.636001 | 1606.007132 | 699.8831246 | 148.684607 | 21.77033809 |
| Liberia | Female | 3697.519856 | 4056.021475 | 4175.535689 | 3182.780276 | 1924.745808 | 886.8969277 | 118.2201418 |
| Libya | Female | 271.311768 | 487.7193354 | 914.4561925 | 769.0097724 | 553.5392967 | 63.11451997 | 8.229725862 |
| Lithuania | Female | 654.3287237 | 1695.018378 | 1859.806196 | 850.0690729 | 193.0759908 | 10.35383205 | 1.290644002 |
| Luxembourg | Female | 59.21456205 | 154.5036517 | 259.1553168 | 200.5280253 | 74.72539152 | 7.531109408 | 2.349273683 |
| Madagascar | Female | 3391.407401 | 3330.121893 | 3276.594567 | 2366.34464 | 1278.575569 | 389.75525 | 50.14105832 |
| Malawi | Female | 3686.717758 | 3890.752492 | 3925.280994 | 3023.846482 | 1505.574768 | 665.0407515 | 85.88325639 |
| Malaysia | Female | 387.1780014 | 1124.734395 | 1765.009619 | 1470.571724 | 529.6587887 | 50.01813736 | 7.03280148 |
| Maldives | Female | 672.529285 | 996.106234 | 1231.792296 | 825.9699021 | 348.5503302 | 34.47850071 | 5.218580816 |
| Mali | Female | 4570.196446 | 6318.801845 | 6145.441783 | 4771.835097 | 2655.131223 | 841.8245476 | 110.3147134 |
| Malta | Female | 260.4454383 | 658.6596664 | 929.9794933 | 534.4587315 | 141.9290138 | 9.095067993 | 1.409352828 |
| Marshall Islands | Female | 993.8398913 | 1085.978548 | 1136.380219 | 824.2331257 | 261.4779238 | 47.17485269 | 7.328121027 |
| Mauritania | Female | 3371.088517 | 4119.176153 | 4776.302287 | 3456.168109 | 1718.188122 | 747.1211505 | 101.0087627 |
| Mauritius | Female | 392.2843033 | 764.5112412 | 1006.433651 | 611.1024187 | 192.7838075 | 14.91959354 | 2.457765546 |
| Mexico | Female | 1829.224366 | 2159.544479 | 1939.000802 | 1164.878163 | 361.6345378 | 36.28268661 | 9.758918458 |
| Micronesia (Federated States of) | Female | 866.9118961 | 1266.998608 | 1507.212072 | 1423.460134 | 399.9523467 | 136.9733639 | 18.83166966 |
| Monaco | Female | 234.4931847 | 553.6499416 | 941.1846274 | 606.1246592 | 179.1924908 | 13.2566153 | 2.108736186 |
| Mongolia | Female | 391.7682145 | 458.5545904 | 675.5486985 | 769.4955049 | 333.3655876 | 34.94105492 | 4.306673814 |
| Montenegro | Female | 396.4127013 | 825.5524883 | 800.7272442 | 454.8408171 | 137.7718159 | 11.94885457 | 1.657155284 |
| Morocco | Female | 499.186856 | 682.8033373 | 996.0356266 | 1030.101474 | 560.8427843 | 83.26469508 | 11.91246844 |
| Mozambique | Female | 4100.856234 | 4574.985004 | 4183.308564 | 3133.334479 | 1756.923705 | 682.7594426 | 88.20176841 |
| Myanmar | Female | 578.5352033 | 899.2034717 | 1080.45675 | 976.7467076 | 517.9341366 | 67.35444143 | 9.269423898 |
| Namibia | Female | 2445.554597 | 3193.100933 | 2714.76404 | 1947.409081 | 771.0429283 | 153.33235 | 21.9680311 |
| Nauru | Female | 1395.446 | 1686.473361 | 2113.341199 | 1298.177183 | 509.8046729 | 116.3867718 | 16.26587889 |
| Nepal | Female | 1108.942733 | 818.9738441 | 549.7776894 | 284.8191523 | 91.41068821 | 47.85057564 | 6.064121749 |
| Netherlands | Female | 86.26486402 | 336.3237389 | 550.9203797 | 340.9074077 | 103.6001901 | 9.323883631 | 1.89129149 |
| New Zealand | Female | 466.1905819 | 932.8741078 | 1326.497812 | 926.0274209 | 285.2657486 | 23.49730915 | 3.289760391 |
| Nicaragua | Female | 679.7187082 | 645.88398 | 675.5177992 | 554.5702934 | 227.0176037 | 30.42418028 | 8.195100971 |
| Niger | Female | 4893.933789 | 7138.323334 | 7012.543559 | 5111.501426 | 2718.684531 | 1036.227863 | 135.0434139 |
| Nigeria | Female | 4368.597958 | 5506.360433 | 5459.254878 | 3655.247346 | 1907.855526 | 635.697378 | 83.44394183 |
| Niue | Female | 923.9926344 | 1260.237502 | 1671.632839 | 1250.375681 | 256.3996038 | 45.72013182 | 6.966616828 |
| North Macedonia | Female | 333.5078001 | 613.8409421 | 558.4038458 | 283.0733537 | 75.95727036 | 6.584163419 | 0.938528595 |
| Northern Mariana Islands | Female | 956.4022633 | 1272.716247 | 1735.316844 | 1091.562868 | 340.4436162 | 22.39815084 | 4.333029406 |
| Norway | Female | 323.6839115 | 1093.352965 | 1423.125749 | 840.9986033 | 232.6088059 | 20.17985308 | 3.575765359 |
| Oman | Female | 431.9745153 | 902.0261605 | 1321.462306 | 1216.096902 | 822.3993121 | 108.8663684 | 13.78137783 |
| Pakistan | Female | 1899.146142 | 2128.970895 | 2135.57112 | 1260.460261 | 607.3075507 | 183.4411519 | 22.56002986 |
| Palau | Female | 928.4283397 | 1253.90006 | 1474.996038 | 1080.816528 | 377.1066117 | 41.75978186 | 6.466736041 |
| Palestine | Female | 688.6511174 | 860.1631293 | 928.3269173 | 669.4024458 | 240.8747011 | 30.92366396 | 4.977947999 |
| Panama | Female | 696.2929308 | 709.9939246 | 760.7663875 | 638.7462913 | 256.6456197 | 25.71926719 | 7.092743034 |
| Papua New Guinea | Female | 1240.53403 | 1249.957652 | 1302.911016 | 1121.327868 | 818.8161388 | 338.0579599 | 44.6718377 |
| Paraguay | Female | 990.650043 | 1169.103653 | 1241.53263 | 960.1632468 | 403.8483304 | 30.04831571 | 5.571929066 |
| Peru | Female | 272.7329836 | 302.9888784 | 335.0961118 | 289.2797592 | 147.8417002 | 15.79776006 | 4.292348308 |
| Philippines | Female | 1113.680679 | 1324.289703 | 1431.318188 | 1190.014653 | 481.4877486 | 69.72446131 | 9.652246935 |
| Poland | Female | 254.3549806 | 524.368529 | 463.5951553 | 240.6548848 | 72.29368163 | 6.551811709 | 1.047949774 |
| Portugal | Female | 75.78994356 | 438.6589008 | 478.9614172 | 398.2508615 | 162.5762486 | 15.57024188 | 2.179887042 |
| Puerto Rico | Female | 761.3739556 | 711.8199037 | 619.1818056 | 396.9641676 | 115.3576299 | 9.239752877 | 2.259937773 |
| Qatar | Female | 452.9297065 | 761.6006296 | 1073.123431 | 921.4817992 | 503.9743359 | 63.16728946 | 8.498102409 |
| Republic of Korea | Female | 25.02127597 | 75.80109411 | 183.225973 | 159.6718501 | 41.20706592 | 2.068857906 | 0.425874452 |
| Republic of Moldova | Female | 1002.61808 | 1043.465874 | 769.6992327 | 378.3381077 | 89.79501228 | 5.724443255 | 0.731296348 |
| Romania | Female | 865.3768951 | 1361.4018 | 1141.161816 | 584.1864169 | 148.6340265 | 10.35835251 | 1.340132643 |
| Russian Federation | Female | 1252.573056 | 1906.716885 | 1767.123199 | 808.7831785 | 200.637456 | 13.71986238 | 1.708811496 |
| Rwanda | Female | 2585.707748 | 4325.334592 | 4223.591222 | 2967.330327 | 1425.158375 | 199.6322273 | 25.66803335 |
| Saint Kitts and Nevis | Female | 659.564589 | 615.5831985 | 655.694394 | 457.169176 | 161.6690453 | 10.12104201 | 2.293352999 |
| Saint Lucia | Female | 613.6842996 | 633.1359999 | 684.5217505 | 563.170277 | 223.3859387 | 14.37642128 | 2.864486401 |
| Saint Vincent and the Grenadines | Female | 859.4934742 | 837.4839032 | 833.7179659 | 771.1720905 | 303.8726832 | 19.37777994 | 3.708899887 |
| Samoa | Female | 1268.690296 | 1869.801205 | 2105.007456 | 1910.445796 | 879.3909776 | 167.2638539 | 22.5690291 |
| San Marino | Female | 123.6903489 | 430.9899558 | 771.9155749 | 608.9059643 | 210.1059143 | 21.8757057 | 3.050357295 |
| Sao Tome and Principe | Female | 2621.642091 | 3026.965338 | 2757.556784 | 2014.450571 | 931.567062 | 163.1929609 | 25.12455966 |
| Saudi Arabia | Female | 363.6329863 | 761.9932114 | 995.9123297 | 931.3849722 | 464.8353848 | 124.1196677 | 15.61943054 |
| Senegal | Female | 2998.934798 | 3934.019758 | 4599.826076 | 3525.260344 | 1775.59079 | 464.0981324 | 61.05498496 |
| Serbia | Female | 244.0495201 | 433.0397044 | 430.7818611 | 253.9651546 | 80.56519357 | 8.854299535 | 1.286600753 |
| Seychelles | Female | 839.4176448 | 1084.504617 | 1264.047171 | 1035.627451 | 446.1278434 | 27.24696304 | 4.289964324 |
| Sierra Leone | Female | 3240.699164 | 4108.482584 | 4454.33845 | 2988.202109 | 1553.138092 | 611.025191 | 80.09518171 |
| Singapore | Female | 109.8816664 | 236.6736845 | 416.5976832 | 323.8926709 | 87.00228876 | 4.678747731 | 0.383626325 |
| Slovakia | Female | 347.3840255 | 620.7507299 | 661.4626822 | 357.7490861 | 101.3219073 | 6.048029647 | 0.862491939 |
| Slovenia | Female | 423.4053751 | 1145.817567 | 1232.127137 | 647.4348284 | 170.0395604 | 11.79932877 | 1.531170149 |
| Solomon Islands | Female | 1381.194855 | 1509.890821 | 1601.21044 | 1353.143193 | 762.9912554 | 218.4237247 | 29.99220911 |
| Somalia | Female | 4480.543248 | 6866.857492 | 6181.109159 | 4813.457866 | 1955.170188 | 748.4128001 | 94.30800061 |
| South Africa | Female | 2386.007384 | 3187.076176 | 1846.22144 | 1179.401474 | 398.7756682 | 35.02921714 | 6.575846439 |
| South Sudan | Female | 5971.159877 | 7175.77999 | 6844.463998 | 5309.363071 | 3634.00424 | 788.5755329 | 101.8702076 |
| Spain | Female | 269.3980823 | 581.1437671 | 794.3448183 | 652.3889473 | 341.2936324 | 46.39930781 | 6.726138965 |
| Sri Lanka | Female | 442.5981865 | 941.2390827 | 1274.316359 | 864.7484635 | 311.8474977 | 37.39559778 | 5.308780848 |
| Sudan | Female | 678.7744589 | 920.7743963 | 1254.053184 | 997.0991217 | 419.0356486 | 109.9131655 | 14.08258344 |
| Suriname | Female | 969.8607935 | 1016.901495 | 986.4347203 | 720.8860578 | 215.8194575 | 22.52661872 | 4.098706032 |
| Sweden | Female | 136.4524629 | 307.4575838 | 396.0441932 | 361.9395923 | 221.782078 | 21.2213551 | 2.782874499 |
| Switzerland | Female | 141.289071 | 405.43542 | 710.6800176 | 570.6411798 | 169.7842068 | 17.18114501 | 2.369382681 |
| Syrian Arab Republic | Female | 383.9398246 | 690.9057626 | 980.220547 | 860.3780628 | 449.0118934 | 43.94841883 | 6.180760276 |
| Taiwan (Province of China) | Female | 70.34255843 | 190.3906155 | 395.4132808 | 306.0336762 | 57.77164945 | 3.996769978 | 0.922156336 |
| Tajikistan | Female | 367.1399585 | 339.3271533 | 439.3550111 | 372.4890055 | 152.6538663 | 28.83966538 | 3.55914725 |
| Thailand | Female | 459.2844422 | 622.5852654 | 757.3579439 | 507.491372 | 163.0341215 | 13.43703732 | 2.22727807 |
| Timor-Leste | Female | 981.6171663 | 1330.133176 | 1807.639241 | 1532.669104 | 897.7134656 | 178.2308654 | 24.12975791 |
| Togo | Female | 2882.083119 | 3724.178292 | 4344.440884 | 3140.705645 | 1590.867238 | 841.2314968 | 108.2406019 |
| Tokelau | Female | 890.3200539 | 1303.965117 | 1881.068284 | 1057.228895 | 540.2719112 | 111.1052935 | 15.28479284 |
| Tonga | Female | 1398.752205 | 2508.560384 | 2962.640063 | 2137.28552 | 708.6861154 | 60.54086556 | 9.862790661 |
| Trinidad and Tobago | Female | 907.0939164 | 1028.110647 | 1047.795095 | 727.5949533 | 265.5705865 | 14.2111916 | 3.139193974 |
| Tunisia | Female | 197.5460414 | 544.7097963 | 863.8814088 | 724.789563 | 349.9810994 | 38.81927339 | 5.148740947 |
| Turkey | Female | 365.9517203 | 999.7037302 | 1182.247098 | 832.1884548 | 273.0080863 | 29.6933466 | 3.989979768 |
| Turkmenistan | Female | 436.5197206 | 679.3592076 | 777.966478 | 570.1834856 | 178.9709662 | 28.88591642 | 3.658874762 |
| Tuvalu | Female | 1219.504519 | 1858.001065 | 1862.069912 | 1417.082133 | 459.3955262 | 83.84414773 | 12.24953376 |
| Uganda | Female | 4130.822526 | 5074.885539 | 4312.592993 | 3325.314214 | 1658.583327 | 408.8818154 | 53.0609096 |
| Ukraine | Female | 1095.761623 | 1221.757167 | 914.0030974 | 471.5115248 | 121.3197391 | 14.47067481 | 1.79171554 |
| United Arab Emirates | Female | 272.401481 | 585.5232947 | 869.6803596 | 797.3562424 | 601.1561377 | 138.0794304 | 16.62789124 |
| United Kingdom | Female | 371.1850694 | 715.8515773 | 941.398521 | 697.3329917 | 220.6441188 | 20.68021939 | 3.142795307 |
| United Republic of Tanzania | Female | 3889.408799 | 4599.698124 | 4678.632007 | 3189.048995 | 1700.887184 | 457.2306088 | 59.79253687 |
| United States Virgin Islands | Female | 1102.395686 | 1462.570565 | 1035.960312 | 1034.217214 | 278.3536896 | 10.21144539 | 2.928925883 |
| United States of America | Female | 666.820927 | 1491.180151 | 1886.317351 | 1075.220829 | 284.1826396 | 22.18830542 | 2.757793932 |
| Uruguay | Female | 858.3626805 | 1078.203385 | 1293.302165 | 968.6231116 | 318.7480665 | 22.61069208 | 2.924276238 |
| Uzbekistan | Female | 501.5103529 | 463.3083612 | 552.1808532 | 344.5734613 | 108.8378085 | 11.4997029 | 1.683644802 |
| Vanuatu | Female | 1457.961629 | 1555.904848 | 1559.841293 | 1246.748026 | 594.0994719 | 121.0720477 | 17.1619875 |
| Venezuela (Bolivarian Republic of) | Female | 832.1852277 | 754.0832606 | 745.2631791 | 597.489218 | 262.0446813 | 38.90224626 | 10.49157633 |
| Viet Nam | Female | 527.198898 | 751.3512701 | 748.7652214 | 462.2704101 | 170.9622079 | 16.53629226 | 2.670503509 |
| Yemen | Female | 1539.030412 | 1729.933361 | 2386.600785 | 2417.744778 | 1122.914646 | 377.98907 | 45.09511218 |
| Zambia | Female | 4298.511854 | 4657.490324 | 4689.973037 | 3465.964377 | 1690.294515 | 425.614635 | 55.05053709 |
| Zimbabwe | Female | 3171.770246 | 3412.481829 | 2994.595672 | 2108.614095 | 833.6936769 | 162.4019535 | 22.90111788 |

**STable 3. Age distribution of DALYs for maternal hypertensive disorders in different countries in 2021.**

| **location** | **sex** | **20-24 years** | **25-29 years** | **30-34 years** | **35-39 years** | **40-44 years** | **45-49 years** | **50-54 years** |
| --- | --- | --- | --- | --- | --- | --- | --- | --- |
| Afghanistan | Female | 617.6388785 | 645.6810454 | 537.2600668 | 515.8094548 | 381.1125143 | 137.3510763 | 5.891117524 |
| Albania | Female | 6.805220652 | 11.09586691 | 8.707382916 | 4.238980376 | 1.057761586 | 0.117359218 | 0.018410452 |
| Algeria | Female | 46.63053161 | 51.17390293 | 60.55455897 | 60.40675172 | 31.84338934 | 6.12182594 | 0.755916666 |
| American Samoa | Female | 100.1478981 | 128.6728559 | 143.744413 | 160.5741374 | 74.51247495 | 21.00288337 | 9.369631748 |
| Andorra | Female | 2.119472576 | 4.962052483 | 7.745344804 | 6.120325959 | 2.077630359 | 0.21494323 | 0.020824917 |
| Angola | Female | 466.243418 | 547.9301918 | 467.1462751 | 616.729269 | 527.4237699 | 311.1480516 | 69.37425161 |
| Antigua and Barbuda | Female | 36.16987654 | 27.79603909 | 23.85198634 | 18.24686496 | 7.630859743 | 1.612731487 | 0.311060457 |
| Argentina | Female | 33.66848329 | 33.57378545 | 30.88291919 | 27.6656185 | 11.13448019 | 2.696316198 | 0.1233717 |
| Armenia | Female | 8.870143477 | 9.294119124 | 5.544050293 | 4.646741845 | 2.134798664 | 0.298491651 | 0.027383949 |
| Australia | Female | 4.469944364 | 8.928289112 | 11.93912341 | 8.702816053 | 2.844951546 | 0.287195122 | 0.030247602 |
| Austria | Female | 2.118337388 | 4.860009933 | 5.276828798 | 3.51402117 | 0.964039816 | 0.190825338 | 0.02681798 |
| Azerbaijan | Female | 15.42430015 | 12.22927528 | 9.900180012 | 6.729809854 | 2.45419611 | 0.279973672 | 0.02874301 |
| Bahamas | Female | 53.55859012 | 42.96733383 | 47.0078092 | 36.52195981 | 12.47948815 | 2.248377761 | 0.340872599 |
| Bahrain | Female | 16.15747928 | 23.36907285 | 23.22233789 | 19.55008827 | 12.4909832 | 2.69068888 | 0.245992289 |
| Bangladesh | Female | 167.5210955 | 127.9831629 | 94.89185231 | 80.06410899 | 48.27285652 | 19.08783983 | 4.809429076 |
| Barbados | Female | 34.2277638 | 25.79392839 | 26.46234071 | 21.59128467 | 8.722325795 | 1.517564166 | 0.253780436 |
| Belarus | Female | 14.683312 | 19.70428818 | 16.20680033 | 7.790052178 | 2.056547572 | 0.140160021 | 0.016723841 |
| Belgium | Female | 5.395942339 | 14.52200379 | 17.10086302 | 9.256320737 | 2.71591088 | 0.30512967 | 0.031962237 |
| Belize | Female | 53.55170357 | 105.6286519 | 67.83643428 | 64.20390388 | 25.51881641 | 4.761606482 | 1.046908445 |
| Benin | Female | 519.8208815 | 577.9339984 | 449.472639 | 279.8275392 | 172.6953736 | 81.01770862 | 8.8948637 |
| Bermuda | Female | 7.184061216 | 9.161987318 | 13.84301498 | 12.35013183 | 4.020823394 | 0.275549294 | 0.023970226 |
| Bhutan | Female | 173.745687 | 137.6951055 | 105.3084873 | 82.86995104 | 45.47178195 | 12.41484494 | 3.286807673 |
| Bolivia (Plurinational State of) | Female | 266.392124 | 312.3421695 | 352.835567 | 336.4430804 | 266.5116611 | 105.845542 | 8.60359679 |
| Bosnia and Herzegovina | Female | 6.217089687 | 9.952961441 | 7.884771291 | 4.045677659 | 0.936680665 | 0.096881939 | 0.01864006 |
| Botswana | Female | 105.6211891 | 86.201903 | 53.05707601 | 33.00907133 | 14.10291633 | 4.524436849 | 0.591999961 |
| Brazil | Female | 59.70446378 | 54.52358166 | 49.41858801 | 38.42368775 | 17.73222356 | 4.399684802 | 0.381114533 |
| Brunei Darussalam | Female | 12.48042696 | 9.399852759 | 30.20159676 | 26.72104316 | 9.676718317 | 1.91910757 | 0.895992572 |
| Bulgaria | Female | 12.98459111 | 11.97660996 | 9.093191104 | 6.259666785 | 1.6797147 | 0.272164399 | 0.057835235 |
| Burkina Faso | Female | 564.8625015 | 547.8728157 | 466.7008361 | 388.6821332 | 228.433714 | 109.2276397 | 9.623182646 |
| Burundi | Female | 539.409792 | 643.1449813 | 629.830762 | 539.7867641 | 411.2944777 | 225.3839147 | 30.78442413 |
| Cabo Verde | Female | 55.0520481 | 74.46027531 | 59.92788944 | 40.30464267 | 15.71635207 | 6.269860007 | 0.67999627 |
| Cambodia | Female | 131.9253597 | 165.2233121 | 153.5759597 | 145.427765 | 91.56108093 | 38.09526982 | 14.12477977 |
| Cameroon | Female | 592.0064217 | 428.4905158 | 339.4865044 | 222.020328 | 132.5378662 | 62.69531307 | 5.465337196 |
| Canada | Female | 3.667143629 | 5.147854277 | 5.235652826 | 3.236660972 | 0.981605918 | 0.322022658 | 0.1313793 |
| Central African Republic | Female | 927.0196508 | 1148.344429 | 824.644598 | 1039.890322 | 780.7418058 | 291.2435324 | 57.546288 |
| Chad | Female | 1332.873935 | 1287.220137 | 943.2606748 | 660.3343695 | 417.5284698 | 170.3209142 | 17.9889402 |
| Chile | Female | 19.63545081 | 23.08363382 | 26.59280643 | 20.50265065 | 8.737940827 | 1.20418395 | 0.074986229 |
| China | Female | 8.116782826 | 10.74140335 | 8.927044544 | 5.87904584 | 2.151946228 | 0.58660986 | 0.233331165 |
| Colombia | Female | 55.5438188 | 42.26386169 | 35.30813964 | 30.75553079 | 12.92513333 | 2.065742806 | 0.205411473 |
| Comoros | Female | 146.0323758 | 424.4589212 | 464.9271606 | 240.7230224 | 155.7620601 | 113.3653082 | 18.334463 |
| Congo | Female | 487.2716206 | 621.6443549 | 486.5747279 | 580.9178762 | 379.4733205 | 237.5631827 | 41.10019651 |
| Cook Islands | Female | 10.720647 | 11.5781481 | 13.10845581 | 11.01645284 | 4.342575641 | 0.725209156 | 0.099749672 |
| Costa Rica | Female | 14.10146941 | 13.69932392 | 15.96032632 | 13.78502149 | 6.359756609 | 1.266726568 | 0.122450588 |
| Croatia | Female | 4.108836896 | 7.286397839 | 5.182045436 | 2.975137255 | 0.749401959 | 0.137779532 | 0.025379206 |
| Cuba | Female | 20.5299821 | 20.51377739 | 16.86210717 | 10.53389047 | 3.948163919 | 0.573162548 | 0.082710172 |
| Cyprus | Female | 2.036316375 | 5.478550482 | 6.848482621 | 4.419154357 | 1.502054402 | 0.340116786 | 0.038615922 |
| Czechia | Female | 6.249822153 | 12.21100238 | 13.37163343 | 7.36098284 | 1.884039854 | 0.150584055 | 0.019120424 |
| Côte d'Ivoire | Female | 579.8497604 | 713.7357395 | 639.4169781 | 513.9018509 | 324.377152 | 207.115787 | 21.72163684 |
| Democratic People's Republic of Korea | Female | 26.46552739 | 35.21237082 | 32.24279006 | 22.39106694 | 7.913914919 | 1.328200621 | 0.290613302 |
| Democratic Republic of the Congo | Female | 687.0311951 | 706.715136 | 535.3062722 | 519.8297306 | 490.5610649 | 247.6096393 | 46.41475783 |
| Denmark | Female | 2.712701499 | 6.691895794 | 6.418687056 | 3.806547386 | 1.247696415 | 0.234034203 | 0.041164507 |
| Djibouti | Female | 833.864082 | 919.6096109 | 799.1759617 | 562.0964757 | 315.3706575 | 119.8875583 | 19.02350112 |
| Dominica | Female | 18.59059927 | 19.01511367 | 17.79761045 | 12.97965763 | 4.8454344 | 0.551897906 | 0.10469546 |
| Dominican Republic | Female | 146.3695481 | 125.7508287 | 109.671224 | 76.99794728 | 34.95244673 | 8.923721773 | 1.251119246 |
| Ecuador | Female | 117.9216305 | 115.6690881 | 120.6157462 | 110.628992 | 49.77101079 | 14.7534102 | 1.490987104 |
| Egypt | Female | 61.32114846 | 74.86911096 | 69.91191638 | 61.36314483 | 24.63175727 | 6.203893718 | 1.137760643 |
| El Salvador | Female | 31.64741354 | 34.1020126 | 31.91972437 | 24.55613411 | 9.437142762 | 1.315690695 | 0.163643508 |
| Equatorial Guinea | Female | 408.7291922 | 439.6303046 | 389.6070842 | 471.5998668 | 429.8937558 | 252.5397084 | 43.79696211 |
| Eritrea | Female | 756.4978514 | 737.8722567 | 615.1967338 | 396.4692841 | 212.9083966 | 65.8941601 | 9.872863309 |
| Estonia | Female | 10.11143781 | 19.38118272 | 21.09123539 | 12.5463765 | 3.917875127 | 0.208933642 | 0.021827457 |
| Eswatini | Female | 137.0391853 | 109.4173682 | 130.0848746 | 134.937412 | 91.42507033 | 85.93496009 | 5.991913449 |
| Ethiopia | Female | 299.4263432 | 355.9633268 | 344.6944511 | 333.2027342 | 281.7010658 | 167.9072931 | 20.06262338 |
| Fiji | Female | 79.82663912 | 97.09633337 | 99.48655608 | 108.8587215 | 55.50681491 | 50.19234109 | 14.54643372 |
| Finland | Female | 4.847931381 | 9.977320469 | 12.09905101 | 7.783723413 | 2.492852232 | 0.247003967 | 0.026796777 |
| France | Female | 5.500953855 | 12.94460234 | 14.50235972 | 9.222342021 | 2.926992637 | 0.325582267 | 0.038017554 |
| Gabon | Female | 288.1455643 | 232.4861172 | 169.7873761 | 178.9336821 | 131.0229891 | 68.27599177 | 8.837614186 |
| Gambia | Female | 675.9058477 | 740.2328421 | 676.649976 | 496.7971478 | 378.7558179 | 324.1812626 | 37.34761715 |
| Georgia | Female | 9.445445951 | 11.28337853 | 11.5004061 | 9.103657591 | 4.687378892 | 0.673121177 | 0.073181604 |
| Germany | Female | 5.085564481 | 12.37392063 | 18.24778049 | 13.2527679 | 3.990552214 | 0.394027465 | 0.046194843 |
| Ghana | Female | 345.3232895 | 276.2129947 | 236.7247309 | 172.1566294 | 80.81534486 | 20.66356798 | 3.519491884 |
| Greece | Female | 3.617668533 | 7.703038657 | 10.38090277 | 6.775739928 | 2.538494225 | 0.5658954 | 0.053272683 |
| Greenland | Female | 7.108358025 | 8.697834653 | 7.18543637 | 3.362642021 | 0.791656199 | 0.089198366 | 0.01377763 |
| Grenada | Female | 164.1055354 | 130.4290327 | 144.8007251 | 112.2017545 | 40.73641992 | 8.665549312 | 1.603204309 |
| Guam | Female | 24.57800807 | 34.39104936 | 52.62390245 | 76.68963618 | 21.98090242 | 8.641091194 | 5.338274417 |
| Guatemala | Female | 204.4891563 | 240.0731982 | 195.0799774 | 176.5534474 | 183.0338672 | 27.91860559 | 2.159012948 |
| Guinea | Female | 914.3838043 | 1085.876198 | 884.3032448 | 676.1595926 | 490.2803393 | 279.260029 | 31.31362522 |
| Guinea-Bissau | Female | 540.0871376 | 602.5034526 | 476.9778573 | 342.8672383 | 191.4653798 | 79.98777291 | 7.700081711 |
| Guyana | Female | 175.3970515 | 152.6957821 | 140.4832624 | 77.15969899 | 33.86218878 | 8.653992875 | 1.505126674 |
| Haiti | Female | 720.092978 | 1107.034547 | 1063.790994 | 610.6055846 | 705.0564026 | 352.1759812 | 28.75195095 |
| Honduras | Female | 141.1738944 | 170.1095595 | 170.5215385 | 168.5058213 | 110.8121508 | 31.6853468 | 4.205355218 |
| Hungary | Female | 6.27244524 | 5.700088089 | 7.349939168 | 3.989530702 | 1.06972482 | 0.109532606 | 0.019296301 |
| Iceland | Female | 4.848188909 | 9.234315317 | 10.57680361 | 6.210043125 | 2.309281601 | 0.361221857 | 0.033882048 |
| India | Female | 118.4232614 | 139.2743985 | 134.2925161 | 109.8761042 | 63.52028521 | 10.37930351 | 6.827845861 |
| Indonesia | Female | 217.6569923 | 227.0301257 | 201.5210865 | 166.8216325 | 110.5181961 | 59.77925992 | 21.042425 |
| Iran (Islamic Republic of) | Female | 11.96527217 | 16.38941458 | 17.58059428 | 13.623625 | 5.743188548 | 0.966240406 | 0.186266949 |
| Iraq | Female | 20.08929813 | 26.03649449 | 33.60942145 | 34.44133974 | 30.30193191 | 12.71337722 | 2.137353681 |
| Ireland | Female | 2.671919822 | 5.596617191 | 8.271484582 | 6.878766916 | 2.440268133 | 0.324720785 | 0.032866733 |
| Israel | Female | 8.160209358 | 14.32639346 | 15.16776421 | 10.62932375 | 3.869019718 | 0.634934189 | 0.053611404 |
| Italy | Female | 2.387261414 | 5.606179554 | 6.866258233 | 5.087258282 | 2.108292957 | 0.404017712 | 0.047625247 |
| Jamaica | Female | 69.91785129 | 74.7034939 | 79.52920323 | 48.34079601 | 19.71964391 | 3.851076045 | 0.687937571 |
| Japan | Female | 3.160083053 | 7.438459313 | 9.664404167 | 9.596384714 | 2.803094542 | 0.130698145 | 0.020976285 |
| Jordan | Female | 12.60584249 | 18.79263018 | 20.7241199 | 17.83660445 | 11.40370414 | 2.824843954 | 0.280203933 |
| Kazakhstan | Female | 22.59131715 | 21.65437776 | 28.96098229 | 27.019878 | 9.901000846 | 1.633096343 | 0.131040706 |
| Kenya | Female | 293.5066491 | 337.287435 | 294.8395427 | 262.270471 | 209.9963714 | 251.3479153 | 40.01402617 |
| Kiribati | Female | 104.3644429 | 132.2152067 | 121.4044577 | 108.8240483 | 51.97560946 | 26.51432549 | 3.893488045 |
| Kuwait | Female | 6.010488584 | 9.721409896 | 10.92763337 | 9.965873237 | 4.786658806 | 0.880245347 | 0.113875767 |
| Kyrgyzstan | Female | 60.70632751 | 58.54963581 | 62.20537451 | 40.42884324 | 15.47799151 | 2.420517176 | 0.267532811 |
| Lao People's Democratic Republic | Female | 117.9451911 | 114.0539062 | 93.7817147 | 80.93132114 | 57.6809567 | 27.47669858 | 12.64215245 |
| Latvia | Female | 10.47474396 | 17.65210695 | 17.35354238 | 10.95091373 | 3.183604617 | 0.210942594 | 0.021743913 |
| Lebanon | Female | 10.61506722 | 14.58974442 | 14.67250018 | 10.78015171 | 4.29012691 | 0.890668464 | 0.072039673 |
| Lesotho | Female | 337.3147003 | 291.1092297 | 255.3960477 | 220.9232215 | 188.1403647 | 134.750071 | 6.252436964 |
| Liberia | Female | 567.7955901 | 265.9207403 | 188.1135935 | 143.5162333 | 93.13051751 | 52.43504895 | 4.494472356 |
| Libya | Female | 16.4643826 | 26.42148931 | 39.00114862 | 40.57262506 | 38.21099864 | 10.04008572 | 0.855380451 |
| Lithuania | Female | 9.492317524 | 20.94767883 | 23.14957593 | 11.65637646 | 2.578540601 | 0.162316645 | 0.015736984 |
| Luxembourg | Female | 2.484652977 | 4.85695994 | 5.745709105 | 2.871115838 | 1.263719614 | 0.164064508 | 0.029587546 |
| Madagascar | Female | 591.6203161 | 561.8698714 | 503.8282155 | 421.468168 | 332.1161267 | 233.0905751 | 42.4622045 |
| Malawi | Female | 386.7443865 | 426.5348696 | 397.0810624 | 436.3245627 | 413.5157451 | 426.5066208 | 96.07175631 |
| Malaysia | Female | 19.46035743 | 33.21824639 | 44.84896069 | 43.8742229 | 24.30123466 | 8.398064363 | 4.073956669 |
| Maldives | Female | 21.38129877 | 25.70231682 | 26.20038626 | 20.22998945 | 8.483497084 | 2.88314455 | 1.238091132 |
| Mali | Female | 653.5067506 | 624.2947679 | 483.3237579 | 301.8697807 | 162.7029988 | 34.42811932 | 3.921592989 |
| Malta | Female | 5.605909835 | 10.05572211 | 17.38344457 | 7.878223201 | 2.910935897 | 0.424903623 | 0.061940661 |
| Marshall Islands | Female | 120.4339408 | 124.599525 | 97.47672897 | 29.60238734 | 4.57455109 | 0.738520035 | 0.070317125 |
| Mauritania | Female | 496.3525416 | 491.2468105 | 489.9028485 | 402.8766966 | 241.8434051 | 221.5516231 | 21.13196836 |
| Mauritius | Female | 20.18002011 | 32.67672271 | 38.63032807 | 28.61499167 | 16.89685327 | 3.253210452 | 2.060532148 |
| Mexico | Female | 56.76386109 | 60.69591561 | 53.56455045 | 34.300962 | 13.53582708 | 3.594218485 | 0.480166367 |
| Micronesia (Federated States of) | Female | 79.01923392 | 40.70028943 | 43.96375827 | 39.70344368 | 13.94266816 | 7.806906554 | 0.969235505 |
| Monaco | Female | 5.673097135 | 10.7917157 | 14.42818686 | 9.29138683 | 2.654747247 | 0.313442145 | 0.031614322 |
| Mongolia | Female | 33.71273455 | 32.64157304 | 34.23095056 | 34.91742353 | 17.53212581 | 2.658462641 | 0.377625816 |
| Montenegro | Female | 5.195986115 | 10.62790467 | 9.490956089 | 5.181613835 | 1.526998213 | 0.138831728 | 0.017954649 |
| Morocco | Female | 54.41264966 | 87.60333 | 93.10336469 | 100.8909788 | 66.25334859 | 19.86182339 | 3.509872224 |
| Mozambique | Female | 399.1129045 | 486.8890999 | 451.5729284 | 461.9331425 | 427.1914255 | 244.1710162 | 39.53839971 |
| Myanmar | Female | 201.2516804 | 206.1635525 | 176.8190114 | 149.2183271 | 78.74011201 | 20.81710427 | 10.25637201 |
| Namibia | Female | 104.8409955 | 148.6976588 | 139.6074987 | 104.513675 | 82.05305147 | 74.5850112 | 5.259003831 |
| Nauru | Female | 217.22483 | 259.3098417 | 268.1047006 | 274.7290943 | 147.2238494 | 125.7147052 | 21.15988048 |
| Nepal | Female | 154.9149851 | 118.1166914 | 94.64938074 | 85.11513757 | 59.62231714 | 20.46642818 | 4.138012169 |
| Netherlands | Female | 4.10690971 | 7.422427934 | 9.427219953 | 5.531037871 | 1.785594422 | 0.309354651 | 0.046009303 |
| New Zealand | Female | 6.520649759 | 12.1070022 | 15.8583379 | 11.35481079 | 3.340181576 | 0.288792246 | 0.024838987 |
| Nicaragua | Female | 48.70993623 | 51.42912832 | 42.95712978 | 39.10176714 | 16.70224526 | 3.582086888 | 0.448090421 |
| Niger | Female | 821.8143196 | 959.3822091 | 783.7790507 | 614.7015003 | 406.3930763 | 240.7783673 | 24.53765586 |
| Nigeria | Female | 629.3209599 | 807.0516685 | 766.3993605 | 638.982475 | 485.2315728 | 216.1578844 | 39.04032704 |
| Niue | Female | 79.03520382 | 64.45893238 | 60.24025366 | 42.89878289 | 13.85920405 | 4.521244427 | 0.469561044 |
| North Macedonia | Female | 9.356534129 | 11.39808708 | 10.32466397 | 5.222980024 | 1.475609224 | 0.166276409 | 0.042277224 |
| Northern Mariana Islands | Female | 35.2822436 | 43.93770229 | 53.74728995 | 50.56405313 | 25.20368686 | 7.707985115 | 2.683759756 |
| Norway | Female | 4.334307931 | 13.85518639 | 16.88617732 | 9.921358929 | 2.505284172 | 0.213482941 | 0.016113611 |
| Oman | Female | 18.52379493 | 31.4474914 | 33.26429352 | 30.41402722 | 20.42495722 | 5.780936969 | 0.949122383 |
| Pakistan | Female | 677.6050832 | 803.5324547 | 667.4040687 | 654.2100036 | 373.8516002 | 105.2659646 | 38.94935524 |
| Palau | Female | 103.6608593 | 43.62946986 | 52.5987142 | 49.5202565 | 20.87337843 | 4.870007786 | 1.595917293 |
| Palestine | Female | 17.99749395 | 18.57852856 | 19.25062204 | 17.97613339 | 8.644204549 | 2.250623204 | 0.274091281 |
| Panama | Female | 52.23737794 | 45.90220995 | 37.97710567 | 37.1554505 | 18.99371162 | 3.60166338 | 0.423704424 |
| Papua New Guinea | Female | 242.9374662 | 217.68866 | 166.2770724 | 148.0793281 | 79.31616752 | 36.76635771 | 3.565811114 |
| Paraguay | Female | 90.70556545 | 84.9484813 | 91.65210972 | 81.67708783 | 41.13638073 | 11.11386413 | 0.972815351 |
| Peru | Female | 119.7934548 | 104.5032067 | 105.2739117 | 86.65080085 | 55.61360241 | 22.88647343 | 2.024074489 |
| Philippines | Female | 139.157397 | 166.5516844 | 169.4720191 | 155.8799693 | 79.78199619 | 19.3029848 | 5.628453388 |
| Poland | Female | 3.402448422 | 7.046670754 | 5.427318446 | 2.76147027 | 0.785516443 | 0.075696431 | 0.010809995 |
| Portugal | Female | 2.838498115 | 7.97701242 | 9.483310496 | 7.210061418 | 2.559042892 | 0.315120652 | 0.042149961 |
| Puerto Rico | Female | 26.64859457 | 23.33811269 | 22.23919728 | 14.55320587 | 5.372500699 | 1.004516001 | 0.223409347 |
| Qatar | Female | 6.328606981 | 10.19858734 | 13.8146202 | 11.28393823 | 6.119111222 | 0.889088708 | 0.103794253 |
| Republic of Korea | Female | 1.06820837 | 2.994887603 | 4.689672661 | 3.143907735 | 0.578578614 | 0.054687053 | 0.005550904 |
| Republic of Moldova | Female | 15.81571277 | 14.3585494 | 10.82762337 | 5.292083483 | 1.464215291 | 0.125056096 | 0.010975805 |
| Romania | Female | 14.88650551 | 20.4272553 | 18.14815931 | 10.80731435 | 2.829052792 | 0.43549511 | 0.16389195 |
| Russian Federation | Female | 18.37201383 | 25.80005149 | 23.45875642 | 11.08583214 | 2.879258269 | 0.226824838 | 0.021392801 |
| Rwanda | Female | 655.7577436 | 732.7375215 | 719.1740865 | 710.5558054 | 650.7943481 | 355.7831914 | 80.8569769 |
| Saint Kitts and Nevis | Female | 238.9209582 | 178.9202487 | 153.11178 | 121.0247503 | 61.20416136 | 12.67385713 | 2.809835198 |
| Saint Lucia | Female | 41.06384918 | 31.45622267 | 29.27796998 | 21.21936501 | 9.117796583 | 1.331227528 | 0.186118535 |
| Saint Vincent and the Grenadines | Female | 105.764547 | 87.1778314 | 59.83494742 | 63.59503464 | 29.16536163 | 5.063480426 | 0.774641905 |
| Samoa | Female | 56.35763799 | 50.89609234 | 49.21469639 | 42.86055977 | 16.63214288 | 4.3656692 | 0.506648492 |
| San Marino | Female | 2.425257338 | 6.011494586 | 9.550871256 | 7.306022074 | 2.420991336 | 0.290760905 | 0.023149442 |
| Sao Tome and Principe | Female | 84.92548403 | 120.7113888 | 100.2879318 | 88.6979476 | 81.10771514 | 57.07061231 | 6.355087203 |
| Saudi Arabia | Female | 15.13734822 | 23.45526204 | 27.11130186 | 28.67795362 | 17.18227194 | 5.292177043 | 0.485798604 |
| Senegal | Female | 529.6360109 | 518.9562664 | 474.0719679 | 340.4591515 | 245.3827584 | 141.1751996 | 15.94857823 |
| Serbia | Female | 7.621448939 | 9.95931341 | 8.222421377 | 4.776901648 | 1.582815442 | 0.20940484 | 0.059644155 |
| Seychelles | Female | 159.6558215 | 193.6398263 | 134.2494715 | 209.2006702 | 81.75383846 | 20.48113367 | 9.827630635 |
| Sierra Leone | Female | 242.7175373 | 333.6565534 | 320.8651812 | 232.0403487 | 200.3462781 | 163.8862497 | 21.9668134 |
| Singapore | Female | 1.797029602 | 3.650441568 | 5.258388436 | 4.029115322 | 0.968599446 | 0.058648911 | 0.003949173 |
| Slovakia | Female | 6.462406192 | 10.11739839 | 9.182360052 | 4.735398047 | 1.288691542 | 0.093546959 | 0.016237683 |
| Slovenia | Female | 5.667926691 | 13.7912927 | 14.36414391 | 7.520819924 | 1.902096047 | 0.144299121 | 0.021143304 |
| Solomon Islands | Female | 419.9529269 | 398.4315064 | 302.8547041 | 229.4870596 | 94.7582008 | 36.689883 | 6.933185889 |
| Somalia | Female | 511.130165 | 582.8166665 | 491.7372832 | 431.4451499 | 330.8257666 | 229.7331563 | 34.05967838 |
| South Africa | Female | 108.296449 | 135.4248009 | 155.8811409 | 129.0714969 | 61.56652228 | 29.76975681 | 1.72047119 |
| South Sudan | Female | 1096.247779 | 1175.46694 | 1032.524346 | 884.1812405 | 664.1592502 | 455.7842021 | 69.9954146 |
| Spain | Female | 4.169701894 | 8.012023222 | 11.10281985 | 8.297945682 | 4.003725159 | 0.573702345 | 0.052724966 |
| Sri Lanka | Female | 22.88814001 | 28.72374002 | 27.89860963 | 19.58544102 | 7.015102649 | 1.328093397 | 0.380167231 |
| Sudan | Female | 220.9167705 | 194.0549992 | 199.8846207 | 223.3321447 | 255.3118716 | 120.1530099 | 39.05072636 |
| Suriname | Female | 169.2308791 | 111.9582905 | 115.0436743 | 85.44874021 | 36.49973702 | 5.684778252 | 0.855329088 |
| Sweden | Female | 4.310781062 | 6.691003373 | 7.942152027 | 5.420672338 | 2.899963226 | 0.301356152 | 0.041040936 |
| Switzerland | Female | 2.425080099 | 6.268984051 | 9.654579949 | 6.881679862 | 1.946821049 | 0.22428852 | 0.017939565 |
| Syrian Arab Republic | Female | 18.04415067 | 23.96810514 | 26.81525274 | 23.74113399 | 13.32785374 | 3.560037018 | 0.339485121 |
| Taiwan (Province of China) | Female | 2.454726285 | 3.675609536 | 6.826382776 | 5.473264358 | 1.170044049 | 0.122114773 | 0.02677904 |
| Tajikistan | Female | 39.53615575 | 47.18602945 | 44.28643452 | 33.58592307 | 14.10004897 | 2.232668939 | 0.234158337 |
| Thailand | Female | 21.98774075 | 27.49437995 | 30.15634798 | 21.82909456 | 10.96738355 | 2.972885822 | 0.982957913 |
| Timor-Leste | Female | 115.7758348 | 136.101173 | 137.796262 | 164.1434514 | 161.7432885 | 95.55986851 | 50.54856681 |
| Togo | Female | 289.1872448 | 306.2277751 | 239.3124278 | 203.620479 | 112.8291701 | 78.32043448 | 8.388860179 |
| Tokelau | Female | 81.52358986 | 65.14272507 | 64.9999773 | 43.6650127 | 18.54564269 | 9.754376343 | 1.038259668 |
| Tonga | Female | 75.33450314 | 64.08787127 | 65.16140149 | 54.57977449 | 26.99244494 | 22.10531775 | 3.101694199 |
| Trinidad and Tobago | Female | 81.43234533 | 93.93324829 | 77.74854626 | 66.88966655 | 28.6194052 | 5.856137331 | 0.91124145 |
| Tunisia | Female | 24.32802034 | 33.99526262 | 37.1535144 | 28.16190801 | 13.17021264 | 2.256878839 | 0.310072563 |
| Turkey | Female | 15.53010287 | 23.67228839 | 26.23255579 | 20.77205843 | 9.566705297 | 2.769497277 | 0.326688486 |
| Turkmenistan | Female | 81.75033466 | 82.27081604 | 92.42247878 | 66.30558091 | 31.99467992 | 2.792347542 | 0.386581387 |
| Tuvalu | Female | 68.99018235 | 82.98631877 | 88.65089092 | 85.64286658 | 38.26404387 | 19.82436396 | 1.461142606 |
| Uganda | Female | 466.2749028 | 520.1888234 | 496.6872056 | 474.3874259 | 411.2784131 | 412.6098962 | 98.60780665 |
| Ukraine | Female | 18.26563722 | 20.88280798 | 13.94869761 | 6.550652047 | 1.645825392 | 0.185952447 | 0.017501855 |
| United Arab Emirates | Female | 11.8920216 | 27.44540997 | 19.34900787 | 14.40551643 | 11.32347407 | 2.585219183 | 0.308329109 |
| United Kingdom | Female | 5.305871839 | 9.673990916 | 11.72128486 | 8.447420835 | 2.538820461 | 0.308876303 | 0.019010792 |
| United Republic of Tanzania | Female | 755.3843141 | 666.5589283 | 528.5062825 | 470.4606597 | 421.7376633 | 396.8848534 | 76.99394265 |
| United States Virgin Islands | Female | 64.94491697 | 39.7007793 | 41.52794239 | 30.71137063 | 11.20834213 | 0.731947205 | 0.144623863 |
| United States of America | Female | 12.99682462 | 24.41099996 | 29.63736663 | 19.28560483 | 6.553547651 | 1.380827858 | 0.246751431 |
| Uruguay | Female | 26.78881081 | 28.92890039 | 25.3812292 | 21.23657863 | 9.126365976 | 1.669500774 | 0.085638303 |
| Uzbekistan | Female | 44.52246447 | 38.23147999 | 40.56777778 | 24.22616845 | 8.898335404 | 1.481374811 | 0.154185988 |
| Vanuatu | Female | 103.9149421 | 118.5349299 | 113.0139585 | 131.9772915 | 78.04202417 | 86.77880719 | 16.34283833 |
| Venezuela (Bolivarian Republic of) | Female | 146.5018755 | 117.5347967 | 113.0583909 | 69.62511961 | 32.56437914 | 6.35497885 | 0.709692426 |
| Viet Nam | Female | 10.96667069 | 14.11211241 | 12.8152957 | 7.795159935 | 2.838434797 | 0.510108319 | 0.135721529 |
| Yemen | Female | 296.5666019 | 325.1822532 | 283.7227397 | 231.045644 | 117.1830894 | 28.53434644 | 3.643081195 |
| Zambia | Female | 450.9833046 | 392.2252215 | 326.5581353 | 221.6477066 | 135.3770524 | 85.9449987 | 9.123806888 |
| Zimbabwe | Female | 416.2556922 | 386.0125822 | 346.4493877 | 325.548371 | 257.5453282 | 206.0607111 | 17.4378996 |

**STable 4. The Top and Bottom three regions of maternal hypertensive disorders in 2021 and trends from 1990 to 2021.**

| Age-standardized DALY rate (95% UI) |  |  | EAPC (95% CI) |  |
| --- | --- | --- | --- | --- |
| Top |  |  |  |  |
|  | Central Sub-Saharan Africa | 255.22 (182.06, 348.26) | Caribbean | 0.75 (0.5296, 0.9614) |
|  | Eastern Sub-Saharan Africa | 191.51 (155.03, 233.27) | High-income North America | -0.32 (-0.5066, -0.1313) |
|  | Caribbean | 126.37 (81.88, 183.60) | Oceania | -0.55 (-0.7322, -0.3587) |
| Bottom |  |  |  |  |
|  | High-income Asia Pacific | 1.90 (1.30, 2.74) | East Asia | -5.36 (-5.6106, -5.1146) |
|  | Central Europe | 2.72 (1.87, 3.95) | North Africa and Middle East | -4.78 (-4.9074, -4.6430) |
|  | Western Europe | 2.88 (1.82, 4.46) | Andean Latin America | -4.19 (-4.4097, -3.9609) |
| ASIR (95% UI) |  |  | EAPC (95% CI) |  |
| Top |  |  |  |  |
|  | Eastern Sub-Saharan Africa | 1408.09 (1198.03, 1626.08) | Eastern Europe | 1.12 (0.6532, 1.5859) |
|  | Central Sub-Saharan Africa | 1323.57 (1098.63, 1609.63) | Western Europe | 0.41 (0.2740, 0.5434) |
|  | Southern Sub-Saharan Africa | 813.53 (681.66, 958.35) | Central Asia | 0.40 (0.1082, 0.7022) |
| Bottom |  |  |  |  |
|  | East Asia | 108.74 (84.21, 140.96) | South Asia | -2.80 (-3.0578, -2.5501) |
|  | High-income Asia Pacific | 154.28 (132.33, 182.35) | High-income Asia Pacific | -1.52 (-1.9896, -1.0518) |
|  | Central Europe | 169.62 (138.88, 210.94) | Central Sub-Saharan Africa | -1.51 (-1.6319, -1.3856) |

**STable 5. The Top and Bottom three countries in 2021 and trends from 1990 to 2021.**

| Age-standardized DALY rate (95% UI) |  |  | EAPC (95% CI) |  |
| --- | --- | --- | --- | --- |
| Top |  |  |  |  |
|  | Wajir | 588.43 (330.70, 873.05) | Guam | 1.78 (1.3228, 2.2419) |
|  | Gilgit-Baltistan | 584.62 (236.82, 1150.04) | American Samoa | 0.50 (0.1057, 0.8860) |
|  | Balochistan | 459.99 (241.31, 759.00) | United Republic of Tanzania | -0.11 (-0.4863, 0.2636) |
| Bottom |  |  |  |  |
|  | Republic of Korea | 0.92 (0.65, 1.24) | Syrian Arab Republic | -7.10 (-8.0072, -6.1814) |
|  | Republic of Singapore | 1.18 (0.67, 1.97) | Lao People's Democratic Republic | -6.70 (-6.8693, -6.5204) |
|  | Republic of Austria | 1.27 (0.84, 1.77) | Republic of Korea | -6.59 (-6.9888, -6.1936) |
| ASIR (95% UI) |  |  | EAPC (95% CI) |  |
| Top |  |  |  |  |
|  | Republic of South Sudan | 2337.94 (2015.37, 2688.94) | Russian Federation | 1.53 (1.0510, 2.0137) |
|  | Republic of the Niger | 2175.70 (1763.51, 2598.50) | Romania | 1.19 (0.5994, 1.7874) |
|  | Republic of Chad | 2138.38 (1783.73, 2544.84) | Georgia | 0.98 (-1.4708, 3.4928) |
| Bottom |  |  |  |  |
|  | Republic of Korea | 34.99 (28.24, 42.44) | Palestine | -3.42 (-3.7854, -3.0440) |
|  | Canada | 39.16 (28.94, 52.54) | Syrian Arab Republic | -2.82 (-2.8709, -2.7608) |
|  | Grand Duchy of Luxembourg | 55.19 (43.29, 70.71) | Saint Lucia | -2.62 (-2.7623, -2.4749) |
